# Disability discrimination and well-being in the United Kingdom: a prospective cohort study

**DOI:** 10.1136/bmjopen-2019-035714

**Published:** 2020-03-12

**Authors:** Ruth A. Hackett, Andrew Steptoe, Raymond P. Lang, Sarah E. Jackson

**Affiliations:** 1 Health Psychology Section, King's College London, London, UK; 2 Behavioural Science and Health, University College London, London, UK; 3 Leonard Cheshire Research Centre, Epidemiology and Public Health, University College London, London, UK

**Keywords:** disability, discrimination, prejudice, stigma, mental health, well-being

## Abstract

**Objectives:**

Disability discrimination is linked with poorer well-being cross-sectionally. The aim of this study was to explore prospective associations between disability discrimination and well-being.

**Design:**

Prospective cohort study.

**Setting:**

The United Kingdom Household Longitudinal Study.

**Participants:**

Data were from 871 individuals with a self-reported physical, cognitive or sensory disability.

**Primary outcome measures:**

Depression was assessed in 2009/10. Psychological distress, mental functioning, life satisfaction and self-rated health were assessed in 2009/10 and 2013/14.

**Results:**

Data were analysed using linear and logistic regression with adjustment for age, sex, household income, education, ethnicity and impairment category. Perceived disability discrimination was reported by 117 (13.4%) participants. Cross-sectionally, discrimination was associated with depression (OR=5.40, 95% CI 3.25 to 8.97) fair/poor self-rated health (OR=2.05; 95% CI 1.19 to 3.51), greater psychological distress (B=3.28, 95% CI 2.41 to 4.14), poorer mental functioning (B=−7.35; 95% CI −9.70 to −5.02) and life satisfaction (B=−1.27, 95% CI −1.66 to −0.87). Prospectively, discrimination was associated with increased psychological distress (B=2.88, 95% CI 1.39 to 4.36) and poorer mental functioning (B=−5.12; 95% CI −8.91 to −1.34), adjusting for baseline scores.

**Conclusions:**

Perceived disability-related discrimination is linked with poorer well-being. These findings underscore the need for interventions to combat disability discrimination.

**Strengths and limitations of this study**

- The use of the prospective United Kingdom Household Longitudinal Study allowed us to examine well-being over a 4-year period.
- The disability discrimination measure took into account several kinds of discriminatory behaviour and included multiple settings where perceived disability discrimination could be encountered.
- Our findings are based on perceptions of disability discrimination rather than objective encounters with disability discrimination.
- Disability discrimination was only assessed at one point in time, meaning our measure does not necessarily reflect pervasive discrimination.

## Introduction

'Disability’ is an umbrella term for long-term impairments, activity limitations and participation restrictions, experienced by an individual with a health condition in interaction with their environment.^1–3^ In the UK, the prevalence of self-reported disability is rising, with 21% of the population reporting a disability in 2017–2018, an increase from 18% in 2007–2008.^4^ This increase is likely driven by population ageing. The majority of disabled people report experiencing difficulties in everyday life. For example, disabled people in Britain have lower educational attainment, are less likely to be employed and are more likely to live in poverty than non-disabled adults,^5^ with knock-on effects regarding access to health and other services.^6^

These practical difficulties experienced by a person with a long-term impairment may be aggravated by and interact with discrimination in the person’s environment. Disability discrimination has been defined as unwanted, exploitative or abusive conduct against disabled people which violates their dignity and security or creates an intimidating or offensive environment.^7^ Although disability is a protected characteristic under equality legislation,^1^ disability discrimination is perceived to be common. In a 2015 population survey of 27 718 adults from 26 European countries, 50% of participants reported disability discrimination to be widespread, a 4% increase from 2012 data. However, the reasons behind this increase are unclear due to the cross-sectional nature of the Eurobarometer data.^8^

Disability discrimination can happen in a variety of settings including on the street, in the workplace and in public venues such as shops or pubs.^9^ Recent British data suggest that disabled people are more likely than non-disabled people to report feeling unsafe when walking alone and to worry about physical attack and theft.^10 11^ Disability is the second most common motivator for hate crime incidents, after race in England and Wales.^12^ In the workplace, several studies suggest that a greater proportion of disabled than non-disabled individuals report experiences of discrimination.^7 13^

A growing body of research has investigated discrimination as a determinant of well-being.^14–16^ Meta-analyses have linked perceived discrimination with depression and psychological distress and with poorer life satisfaction and self-rated health.^14–16^ However, disability discrimination was not assessed in two of these meta-analyses and the majority of previous working has focused on racism.^14 15^ In the most recent pooled analysis of 328 studies, of which only 8 studies concerned physical illness discrimination and disability discrimination,^16^ the combined category of physical illness/disability discrimination was associated with greater psychological distress and lower self-esteem.

Several cross-sectional studies have assessed perceived disability discrimination alone in relation to well-being outcomes. One study of 229 individuals with an intellectual disability in England found that self-reported stigma was associated with a greater number of depression and anxiety symptoms.^17^ Two analyses of the Australian Survey of Disability, Ageing and Carers (n=9655 and n=6183, respectively) linked disability discrimination with greater psychological distress.^18 19^ This association was similarly observed in a Swedish general population survey.^20^ Research has also linked perceived disability discrimination with lower life satisfaction in Canadian adolescents,^21^ Korean women with severe disabilities^22^ and Israeli nationals with physical disabilities.^23^ Perceived disability discrimination has been associated with poorer self-rated health cross-sectionally in four studies^19 24–26^ including a general population analysis of 52 458 individuals, from the European Social Survey.^25^

Cross-sectional correlations are difficult to interpret: perception of discrimination may result in emotional distress, but it is also possible that emotional distress leads to alterations in how people interpret social interactions with others. To date, only one study has assessed prospective associations between perceived disability discrimination and well-being.^27^ In an analysis of older adults (≥50 years) participating in the US-based Health and Retirement Study (HRS), perceived disability discrimination was associated with poorer life satisfaction, self-rated health and greater loneliness over 4 year follow-up.

Overall, previous research has been dominated by cross-sectional studies, precluding the assessment of the temporal relationship between perceived disability discrimination and well-being outcomes. No longitudinal studies have compared people with a disability who do or do not report discrimination and well-being outcomes. To address these limitations, this study aimed to investigate cross-sectional and prospective associations between perceived disability discrimination and well-being in a UK population cohort.

## Methods

### Study population

This study uses data from Understanding Society: The UK Household Longitudinal Study (UKHLS).^28^ The overarching purpose of UKHLS is to provide high-quality longitudinal data about the health, work, education, income, family and social life of the UK population.^29^ Data collection began in 2009/10 (wave 1) with follow-ups annually. The current study uses data from waves 1 (2009/10) and 5 (2013/14). These data were collected through face-to-face interview via computer-aided personal interview and self-completion paper questionnaires and from wave 3 via computer administered self-interview. The UKHLS comprises a representative general population probability sample of UK households, in addition to an ethnic minority boost sample.^29 30^ The general population sample is based on proportionality stratified clustered samples of residential addresses in England, Scotland and Wales. In Northern Ireland, an unclustered systematic random sample of domestic addresses was selected. The ethnic minority boost was selected from high concentration ethnic minority areas, where 80% of the UK’s five major ethnic minorities live.^29^

Our data come from the ‘extra 5 min sample’ of over 8000 participants who had an additional 5 min of questions on issues pertinent to ethnicity research including discrimination. This sample comprises mostly ethnic minorities (n=6722) who were drawn from the ethnic minority boost along with a comparison group of white participants (n=1428).^30^ We restricted our sample to those who responded to the disability discrimination questions (n=4788) with a self-reported disability (n=871). Self-reported disability was based on a positive response the question “*Do you have any health problems or disabilities which mean you have substantial difficulties with any of the following areas of your life”* across any of the 12 types of difficulty assessed. These included issues with manual dexterity and mobility, problems with memory or the ability to concentrate, difficulties with learning and understanding as well as hearing and sight impairments. The response rates for the UKHLS general population sample and the ethnic minority boost at wave 1 were 81.8% and 72.4%, respectively.^30^ The response rate for the ‘extra 5 min sample’ was 42.5%. At wave 5, there was loss to follow-up (n=431), leaving a follow-up sample size of 440 participants. Our definition of disability did not include mental health-related impairments.

### Perceived disability discrimination

To assess discrimination, participants were asked whether in the past 12 months they (a) felt unsafe, (b) avoided going to or being in, (c) had been insulted, called names, threatened or shouted at or (d) had been physically attacked, in seven different settings: 1) at school/college/work, 2) on public transport, 3) at or around bus or train stations, 4) in a taxi, 5) public buildings such as shopping centres or pubs, 6) outside on the street, in parks or other public places or 7) at home. If they answered yes to any one of these questions, a follow-up question asked them to choose a reason from a list of categories including disability, sex and ethnicity among others. It was possible to choose multiple settings and attributions for the perceived discrimination. Those who attributed any experience of discrimination to disability are treated as cases of perceived disability discrimination.

### Outcome variables

Self-reported doctor-diagnosed clinical depression was measured at wave 1 (2009/10) with responses coded as yes/no. Depression was not analysed longitudinally due to a lack of incident cases. All other outcomes were assessed at waves 1 (2009/10) and 5 (2013/14). Psychological distress was measured using the General Health Questionnaire (GHQ)-12,^31^ which involved ratings of 12 statements including whether the individual had “*Been able to enjoy your normal day to day activities*” or whether they ‘*felt constantly under strain*’ with response options of 0=‘no’ and 1=‘yes’. Total scores range from 0 (least distressed) to 12 (most distressed). The 12-item Short-Form Health Survey (SF-12) mental component summary score was used to measure limitations caused by emotional, mental health and social functioning issues.^32^ Items included ratings of feelings experienced over the past 4 weeks such as “*Have you felt downhearted or blue*?” or *“Accomplished less than you would like”*. Overall scores were derived using standard methods ranging from 0 (low functioning) to 100 (high functioning).^33^ Life satisfaction was assessed using one item asking participants how satisfied they were with their ‘life overall’, with scores ranging from 1 (completely dissatisfied) to 7 (completely satisfied). Self-rated health was assessed using a single item: “*Would you say your health is…poor/fair/good/very good/excellent?”* In keeping with previous investigations,^34 35^ self-rated health was dichotomised with 0 being ‘good/very good/excellent’ and 1 meaning ‘poor/fair’.

### Covariates

A number of covariates (assessed at wave 1) that are likely relevant to perceived disability discrimination and well-being were selected a priori for inclusion in our analyses. Age in years was entered as a continuous variable, as there may be age differences in reports of discrimination^36^ and in well-being outcomes.^37^ Sex was included as a binary variable (male/female) based on previous literature demonstrating sex differences in the impact of discrimination on health.^38^ Income and education were included as covariates as there may be socioeconomic differences in the perception of discrimination and in well-being outcomes.^36 39^ Equivalised monthly household income was calculated by dividing total household net income by the modified Organization for Economic Cooperation and Development equivalence scale to adjust for the effects of household size and composition.^40^ Income was entered as a continuous variable in our models. Education was included as a 3-level categorical variable: 1 ‘university degree’, 2 ‘high school qualification’ and 3 ‘no qualification’. As our sample was ethnically diverse, we included ethnicity as a 4-level variable with 1 being ‘white’ including those of white British, white Irish and any other white background, 2 being ‘south Asian’ including Indian, Pakistani and Bangladeshi individuals, 3 being ‘black’ including black African and black Caribbean participants and 4 being ‘other’ including individuals from Chinese and mixed backgrounds. There were four categories of impairment measured in the study: ‘physical’ disability which included difficulties with manual dexterity and mobility; ‘cognitive’ disability including problems with memory or the ability to concentrate, learn and understand; ‘sensory’ disability including hearing (apart from using a standard hearing aid) and sight impairments (apart from wearing standard glasses) and ‘other’ which encompassed reports of unspecified disability not captured in the other categories.

### Statistical analyses

We compared the characteristics of those who did and those who did not report disability discrimination at wave 1 using χ^2^ tests for categorical variables and independent samples t-tests for continuous variables. Associations between perceived disability discrimination and the various well-being measures were assessed using linear regression for continuous outcomes and logistic regression for categorical outcomes. For cross-sectional analyses, depression, psychological distress (GHQ-12), SF-12 mental component score, life satisfaction and self-rated health at wave 1 (2009/10) were the outcome variables. For prospective analyses, psychological distress (GHQ-12), SF-12 mental component score, life satisfaction and self-rated health at wave 5 (2013/14) were the outcomes. Age, sex, household income, education, ethnicity and disability type at wave 1 were controlled for in all analyses. Baseline (wave 1) scores/status on the relevant well-being variable was included as an additional covariate in prospective analyses. Only those with complete case information at wave 1 (n=871) and wave 5 (n=440) were included in the analyses. Results from linear regression analyses are presented as unstandardised B and 95% CIs. Results from logistic regression analyses are presented as ORs and 95% CI. All analyses were unweighted and conducted using SPSS V.24.

### Sensitivity analyses

We carried out three sensitivity analyses. In our first, we assessed whether those who were lost to follow-up (n=431) differed from those who provided data at both waves (n=440). We tested whether this impacted the results by conducting the cross-sectional analyses (wave 1) including only those who provided follow-up data at wave 5. We carried out our second sensitivity analysis to test the possibility that one of the four types discriminatory behaviour contributing to the measure of perceived disability discrimination (ie, feeling unsafe, avoiding somewhere, being insulted or being physically attacked) was driving the results. We tested this cross-sectionally and prospectively by repeating our analyses removing each type of discriminatory behaviour in turn. In our third sensitivity analysis, we tested whether the prospective results from our complete case analysis at wave 5 (n=440) were similar when missing outcome information was imputed for those participants lost to follow-up (n=431).

## Results

A total of 871 participants were included in the study and of these 117 (13.4%) reported perceived disability discrimination. Disability discrimination was the most commonly reported form of discrimination in the sample, followed by age discrimination (4.3%), sex discrimination (3.9%), ethnicity discrimination (3.8%), religious discrimination (2.2%) and discrimination on the basis of sexual orientation (0.5%). Of the categories of disability discrimination assessed, the most commonly reported was feeling unsafe (86.1%; 95% CI 79.48 to 92.74), followed by avoiding somewhere (72.8%; 95% CI 64.08 to 81.55), being insulted (23.5%; 95% CI 14.33 to 32.73) and being physically attacked (2.8%; 95% CI 0.04 to 5.98). The most common settings in which disability discrimination was reported were on the street (77.8%; 95% CI 70.13 to 85.42), in public buildings such as shops or pubs (59.8%; 95% CI 50.81 to 68.84), on public transport (51.3%; 95% CI 42.09 to 60.47) and at or around bus or train stations (40.2%; 95% CI 31.16 to 49.19). A quarter of participants reported experiencing disability discrimination at home (25%; 95% CI 17.61 to 33.67). Perceived disability discrimination was less frequently reported in school or workplace settings (12.8%; 95% CI 6.67 to 18.97) or in taxis (12%; 95% CI 6 to 17.93). The prevalence of the various types of perceived disability discrimination and the settings in which the discrimination occurred for different types of disability can be found in online supplementary table 1. There were no statistically significant differences between people with different types of disability in discrimination type or discrimination setting.

10.1136/bmjopen-2019-035714.supp1 Supplementary data

The baseline characteristics of the sample are displayed in table 1. The group who reported disability discrimination were younger on average (48.29±14.89 years) than those who did not report discrimination (53.42±16.56 years). They were more likely to be white (27.4% vs 20.3%) and to be better educated than those who did not report discrimination, with a greater proportion holding university degrees (28.2% vs 22.7%). Physical disability was most common in those who did not perceive discrimination (46.6%), whereas other unspecified disabilities (65%) were most frequently reported by those who perceived discrimination.

**Table 1:** Associations between perceived disability discrimination and sociodemographic factors (wave 1) and well-being measures (waves 1 and 5)

	Overall sample (n=871)	No perceived discrimination (n=754)	Perceived discrimination (n=117)	P value
Age (years)	52.73 (16.43)	53.42 (16.56)	48.29 (14.89)	0.001
17–34	120 (13.7%)	100 (13.3%)	20 (17.1%)	
35–44	172 (19.7%)	149 (19.8%)	23 (19.7%)	
45–54	184 (21.1%)	150 (19.9%)	34 (29.1%)	
55–64	157 (18.0%)	134 (17.8%)	23 (19.7%)	
65+	238 (27.3%)	221 (29.3%)	17 (14.5%)	
Sex (% men)	388 (44.5%)	334 (44.3%)	54 (46.2%)	0.707
Household income (£)	1118.42 (902.54)	1123.28 (930.47)	1087.07 (698.65)	0.687
0–499	117 (13.4%)	105 (13.9%)	12 (10.3%)	
500–999	369 (42.4%)	314 (41.6%)	55 (47.0%)	
1000–1499	216 (24.8%)	189 (25.1%)	27 (23.1%)	
1500–1999	90 (10.3%)	77 (10.2%)	13 (11.1%)	
2000+	79 (9.1%)	69 (9.2%)	10 (8.5%)	
Education (% yes)				0.003
University degree	204 (23.4%)	171 (22.7%)	33 (28.2%)	–
School qualification	342 (39.3%)	285 (37.8%)	57 (48.7%)	–
No qualification	325 (37.3%)	298 (39.5%)	27 (23.1%)	–
Ethnicity				0.002
White	185 (21.2%)	153 (20.3%)	32 (27.4%)	–
South Asian	369 (42.4%)	333 (44.2%)	36 (30.8%)	–
Black	189 (21.7%)	168 (22.3%)	21 (17.9%)	–
Other	128 (14.7%)	100 (13.3%)	28 (23.9%)	–
Disability type				<0.001
Physical	367 (42.1%)	351 (46.6%)	16 (13.7%)	
Sensory	95 (10.9%)	84 (11.1%)	11 (9.4%)	
Cognitive	87 (10.0%)	73 (9.7%)	14 (12.0%)	
Other	322 (37.0%)	246 (32.6%)	76 (65.0%)	
Well-being measures (wave 1)				
Depression	115 (13.2%)	65 (8.7%)	50 (42.7%)	<0.001
Psychological distress	3.43 (3.85)	2.85 (3.49)	6.65 (4.18)	<0.001
SF-12 mental	43.87 (12.61)	45.16 (12.22)	35.72 (12.02)	<0.001
Life satisfaction	4.49 (1.71)	4.71 (1.63)	3.29 (1.67)	<0.001
Fair/Poor self-rated health	620 (71.2%)	525 (69.6%)	95 (81.2%)	0.010
Well-being measures (wave 5)				
Psychological distress	3.33 (3.99)	2.86 (3.82)	5.98 (3.91)	<0.001
SF-12 mental	44.38 (12.20)	45.79 (11.62)	36.47 (12.51)	<0.001
Life satisfaction	4.48 (1.61)	4.62 (1.57)	3.67 (1.64)	<0.001
Fair/Poor self-rated health	276 (62.7%)	235 (61.0%)	41 (74.5%)	0.053

Data are presented as means (SD) and n (%). Percentages are valid per cent.

SF-12, 12-Item Short-Form Health Survey.

### Cross-sectional associations between perceived disability discrimination and well-being

Our findings suggest that individuals who perceived disability discrimination were significantly more likely to report a diagnosis of clinical depression (OR=5.40; 95% CI 3.25 to 8.97, p<0.001) and were more likely to rate their health as fair/poor (OR=2.05; 95% CI 1.19 to 3.51, p=0.009) than those who did not perceive disability discrimination, independent of covariates (first panel table 2). Those who reported discrimination also had significantly higher levels of psychological distress (B=3.28, 95% CI 2.41 to 4.14, p<0.001), poorer mental functioning on the SF-12 (B=−7.35; 95% CI −9.70 to −5.02, p<0.001) and lower life satisfaction (B=−1.27, 95% CI −1.66 to −0.87, p<0.001), than those who did not report discrimination.

**Table 2:** Cross-sectional and prospective associations between perceived disability discrimination and well-being outcomes

	Depression	Psychological distress	SF-12 mental	Life satisfaction	Fair/Poor self-rated health
	OR (95% CI)	B (95% CI)	B (95% CI)	B (95% CI)	B (95% CI)
**Wave 1**					
No perceived discrimination	1 (reference)†	Reference‡	Reference§	Reference¶	1 (reference)††
Perceived discrimination	5.40 (3.25 to 8.97)***	3.28 (2.41 to 4.14)***	−7.35 (−9.70 to −5.02)***	−1.27 (−1.66 to −0.87)***	2.05 (1.19 to 3.51)**
**Wave 5**					
No perceived discrimination Perceived discrimination	–	Reference‡‡	Reference§§	Reference¶¶	1 (reference)†††
	–	2.88 (1.39 to 4.36)***	−5.12 (−8.91 to −1.34)**	−0.53 (−1.18 to 0.11)	1.29 (0.59 to 2.83)

All analyses are adjusted for age, sex, household income, education, ethnicity and disability type. Prospective analyses are additionally adjusted for baseline well-being status/score.

Possible scores on the psychological distress scale range from 0 to 12, SF-12 mental component scale range from 0 to 100 and the life satisfaction scale scores range from 0 to 7.

*p<0.05.

† n= 751 for the no perceived discrimination group; n= 117 for the perceived discrimination group.

‡ n= 454 for the no perceived discrimination group; n= 82 for the perceived discrimination group.

§ n= 742 for the no perceived discrimination group; n= 117 for the perceived discrimination group.

¶ n= 454 for the no perceiveddiscrimination group; n= 84 for the perceived discrimination group.

**p<0.01.

††n= 754 for the no perceived discrimination group; n= 117 for the perceived discrimination group.

‡‡ n= 177 for the no perceived discrimination group; n= 31 for the perceived discrimination group.

§§ n= 239 for the no perceived discrimination group; n= 43 for the perceived discrimination group.

¶¶ n= 171 for the no perceived discrimination group; n= 34 for the perceived discrimination group

***p <0.001.

††† n= 385 for the no perceived discrimination group; n= 55 for the perceived discrimination group.

SF-12, 12-Item Short-Form Health Survey.

### Prospective associations between perceived disability discrimination and well-being

In prospective analyses (second panel table 2), those who reported perceived disability discrimination at wave 1 had higher levels of psychological distress 4 years later at wave 5 than those who did not report discrimination, independent of covariates and baseline psychological distress (B=2.88, 95% CI 1.39 to 4.36, p<0.001). We detected a prospective association between perceived disability discrimination at wave 1 and poorer SF-12 mental functioning at wave 5 (B=−5.12; 95% CI −8.91 to −1.34, p=0.008). Those who reported disability discrimination at wave 1 had slightly lower life satisfaction (means=4.14 vs 4.67) and a greater proportion rated their health as fair/poor (67.3% vs 62.1%) than those who did not report discrimination at follow-up (wave 5). However, these differences did not reach statistical significance.

### Sensitivity analyses

In the first sensitivity analysis (table 3), cross-sectional findings for those who provided complete data at wave 5 were similar to the full-sample at wave 1. The demographic characteristics of those lost to follow-up were similar to those of complete cases (table 4). Only education differed significantly between the groups, with those who provided complete data at wave 5 more likely to hold a degree (27.0%) than those lost to follow-up (19.7%).

**Table 3:** Cross-sectional and prospective associations between perceived disability discrimination and well-being outcomes (complete cases at wave 5)

	Psychological distress†	SF-12 mental‡	Life satisfaction§	Fair/Poor self-rated health¶
	B (95% CI)	B (95% CI)	B (95% CI)	B (95% CI)
**Wave 1**				
No perceived discrimination	Reference	Reference	Reference	1 (reference)
Perceived discrimination	2.65 (1.21 to 4.08)***	−7.20 (−11.01 to −3.39)***	−1.27 (−1.91 to −0.63)***	2.66 (1.16 to 6.08)*
**Wave 5**				
No perceived discrimination Perceived discrimination	Reference	Reference	Reference	1 (reference)
	2.88 (1.39 to 4.36)***	−5.12 (−8.91 to −1.34)**	−0.53 (−1.18 to 0.11)	1.29 (0.59 to 2.83)

All analyses are adjusted for age, sex, household income, education, ethnicity and disability type. Prospective analyses are additionally adjusted for baseline well-being status/score.

Possible scores on the psychological distress scale range from 0 to 12, SF-12 mental component scale range from 0 to 100 and the life satisfaction scale scores range from 0 to 7.

*p<0.05.

†n= 177 for the no perceived discrimination group; n= 31 for the perceived discrimination group.

‡n= 239 for the no perceived discrimination group; n= 43 for the perceived discrimination group.

§n= 171 for the no perceived discrimination group; n= 34 for the perceived discrimination group.

¶n= 385 for the no perceived discrimination group; n= 55 for the perceived discrimination group.

**p<0.01, ***p <0.001

SF-12, 12-Item Short-Form Health Survey.

**Table 4:** Participant characteristics at wave 1 (2009/10) of complete cases and those lost to follow-up

	Lost to follow-up (n=431)	Complete cases (n=440)	P value
Age (years)	52.26 (17.08)	53.19 (15.76)	0.407
17–34	25 (5.8%)	12 (2.7%)	
35–44	46 (10.7%)	37 (8.4%)	
45–54	79 (18.4%)	93 (21.1%)	
55–64	91 (21.2%)	93 (21.1%)	
65+	189 (44.0%)	205 (46.6%)	
Sex (% men)	200 (46.4%)	188 (42.7%)	0.275
Household income (£)	1101.26 (1037.11)	1135.22 (748.46)	0.579
0–499	69 (16.0%)	48 (10.9%)	
500–999	175 (40.6%)	194 (44.1%)	
1000–1499	113 (26.2%)	103 (23.4%)	
1500–1999	42 (9.7%)	48 (10.9%)	
2000+	32 (7.4%)	47 (10.7%)	
Education (% yes)			0.024
University degree	85 (19.7%)	119 (27.0%)	–
School qualification	171 (39.7%)	171 (38.9%)	–
No qualification	175 (40.6%)	150 (34.1%)	–
Ethnicity			0.213
White	88 (20.4%)	97 (22.0%)	–
South Asian	172 (39.9%)	197 (44.8%)	–
Black	105 (24.4%)	84 (19.1%)	–
Other	66 (15.3%)	62 (14.1%)	–
Disability type			0.189
Physical	166 (38.5%)	201 (45.7%)	
Sensory	51 (11.8%)	44 (10.0%)	
Cognitive	47 (10.9%)	40 (9.1%)	
Other	167 (38.7%)	155 (35.2%)	
Mental well-being			
Psychological distress	3.46 (3.85)	3.41 (3.86)	0.874
SF-12	43.72 (12.93)	44.01 (12.30)	0.741
Life satisfaction	4.40 (1.75)	4.56 (1.69)	0.306
Self-rated health (% fair/poor)	319 (74.0%)	301 (68.4%)	0.068

Data are presented as means (SD) and n (%).

*Complete cases are defined as those who were present at wave 1 and provided data on at least one well-being measure at wave 5.

SF-12, 12-Item Short-Form Health Survey.

In the second sensitivity analysis, removing each of the discriminatory behaviours from the measure of discrimination in turn did not alter the cross-sectional results (table 5). Prospectively, the association between perceived disability discrimination and increased psychological distress remained the same regardless of the type of discriminatory behaviour removed from the measure. For SF-12 mental functioning, the association was fairly robust to the type of discriminatory behaviour, but was slightly attenuated when ‘feeling unsafe’ was removed from the discrimination variable (p=0.058). Again, no significant prospective associations were detected for life satisfaction and self-rated health.

**Table 5:** Sensitivity analysis: perceived disability discrimination measure excluding each discriminatory behaviour in turn

Cross-sectional analyses (wave 1)		Model 1	Model 2	Model 3	Model 4
Depression	OR (95% CI)	4.41 (2.55 to 7.60)***	5.24 (3.06 to 8.98)***	4.80 (2.92 to 7.88)***	5.41 (3.26 to 8.98)***
Psychological distress	Coeff. (95% CI)	3.64 (2.68 to 4.60)***	3.28 (2.33 to 4.23)***	3.13 (2.26 to 4.01)***	3.27 (2.41 to 4.14)***
SF-12 mental	Coeff. (95% CI)	−6.63 (−9.29 to −3.97)***	−7.61 (−10.15 to −5.08)***	−7.53 (−9.89 to −5.18)***	−7.35 (−9.68 to −5.02)***
Life satisfaction	Coeff. (95% CI)	−1.23 (−1.69 to −0.76)***	−1.45 (−1.88 to −1.02)***	−1.24 (−1.64 to −0.85)***	−1.27 (−1.66 to −0.87)***
Fair/Poor self-rated health	OR (95% CI)	1.92 (1.04 to 3.53)*	2.39 (1.32 to 4.33)**	2.21 (1.28 to 3.81)**	2.04 (1.19 to 3.50)**
**Prospective analyses (wave 5**)		**Model 1**	**Model 2**	**Model 3**	**Model 4**
Psychological distress	Coeff. (95% CI)	2.78 (1.14 to 4.41)***	2.69 (1.15 to 4.24)***	2.89 (1.45 to 4.33)***	2.88 (1.39 to 4.36)***
SF-12 mental	Coeff. (95% CI)	−4.33 (−8.81 to 0.14)	−4.90 (−8.86 to −0.94)**	−5.94 (−9.59 to −2.28)**	−5.13 (−8.91 to −1.34)**
Life satisfaction	Coeff. (95% CI)	−0.39 (−1.14 to 0.36)	−0.37 (−1.06 to 0.32)	−0.39 (−1.02 to 0.25)	−0.53 (−1.18 to 0.11)
Fair/Poor self-rated health	OR (95% CI)	1.23 (0.50 to 3.03)	1.30 (0.57 to 2.95)	1.31 (0.61 to 2.83)	1.29 (0.59 to 2.83)

All analyses are adjusted for age, sex, household income, education, ethnicity and disability type. Prospective analyses are additionally adjusted for baseline well-being status/score.

Model 1 excludes ‘*felt unsafe at some place’* from the measure of perceived disability discrimination; model 2 excludes ‘*avoided some place’*; model 3 excludes ‘*was insulted at some place’* and model 4 excludes ‘*was attacked at some place’*.

Possible scores on the psychological distress scale range from 0 to 12, SF-12 mental component scale range from 0 to 100 and the life satisfaction scale scores range from 0 to 7.

^*^P<0.05, **p<0.01, ***p<0.001.

Coeff, unstandardised B coefficient.

In our final sensitivity analysis (online supplementary table 2), we repeated the prospective analyses with imputation for missing outcome information of participants lost to follow-up (n=431). The prospective relationship between perceived disability discrimination and poor SF-12 mental functioning remained (p=0.034). However, there was no longer a statistically significant prospective association between reported discrimination and psychological distress (p=0.128).

## Discussion

In a sample of UK-based participants with self-reported disability, perceived discrimination was associated with higher prevalence of depression, greater psychological distress and poorer mental functioning, life satisfaction and self-rated health. Prospectively, disability discrimination was associated with increased psychological distress and worse mental functioning 4 years later. Our results were robust to adjustment for a range of covariates and were not driven by any specific kind of discriminatory behaviour. No significant prospective relationships with life satisfaction and self-rated health were observed.

Previous literature has been dominated by cross-sectional studies. To our knowledge, only one previous study has investigated the prospective association between disability discrimination and well-being outcomes. In this analysis of US adults from the HRS cohort, perceived disability discrimination was associated with poorer life satisfaction and self-rated health over 4-year follow-up.^27^ In the current study, we observed poorer mental functioning and greater psychological distress 4 years later in those who reported disability discrimination, taking into account baseline scores on these variables. We failed to detect a significant association between perceived discrimination and life satisfaction or self-rated health at follow-up. Although on average, those who perceived disability discrimination in our sample had poorer life satisfaction and were more likely to rate their health as fair/poor at follow-up than those who did not perceive discrimination, these differences did not reach statistical significance. One reason for the divergence in findings between our study and the HRS analysis^27^ may be study design. We limited our analyses to those with self-reported disability, whereas in the HRS study associations between well-being and disability discrimination were assessed across the entire sample. Our analysis offers more precision in the assessment of the relationship between disability discrimination and well-being outcomes, by directly comparing people with disability who did and did not perceive discrimination. Another possibility for these null findings may be that significant associations between discrimination and life satisfaction and self-rated health do not become apparent until older adulthood, perhaps allowing for repeated exposures to disability discrimination. However, this assertion remains to be tested. Another potential explanation is that the impact of ongoing disability discrimination on life satisfaction and self-rated health in our sample had already become apparent at the time of the baseline survey, limiting the scope for further decline.

Our study adds to the cross-sectional literature linking perceived disability discrimination and poorer well-being outcomes by demonstrating associations in a community sample of disabled people living in the UK. Our results extend the findings of an earlier study linking stigma and depression in those with intellectual disability,^17^ by establishing this relationship in a sample with a broader range of disability. In keeping with previous studies, we observed greater psychological distress^18–20^ and poorer life satisfaction^21–23 27^ in those who reported disability discrimination. Our study adds to this existing evidence by demonstrating this link in a UK-based sample for the first time. Similar to earlier work from Australian, European and North American samples,^19 24 27 41^ we observed a relationship between perceived disability discrimination and poorer self-rated health. Cross-sectional studies cannot determine whether perceived disability discrimination predicts poor mental well-being, or whether perceptions of discrimination are an indicator of psychological distress. Our prospective findings therefore add to the field in establishing that perceived disability discrimination predicts psychological distress and poorer mental functioning, net of baseline associations, so has negative implications for future well-being.

This is an observational study and longitudinal analyses do not necessarily imply causality. There could be unmeasured factors responsible for the associations that emerged. Nevertheless, with regard to the pathways linking perceived disability discrimination and well-being, there are several possibilities that could explain our results. One mechanism could be that perceptions of disability discrimination in healthcare settings serve to impede access to health services. An analysis of HRS found that reports of frequent discrimination in healthcare settings were predictive of new or worsened disability over 4-year follow-up.^42^ Quantitative^38 43^ and qualitative^44^ evidence suggests that those who perceive disability discrimination are less likely to seek healthcare. However, there may be sex differences in this association, with a Swedish study only detecting a relationship between disability discrimination and healthcare avoidance in women.^38^ However, no interaction between sex and perceived discrimination was detected in the current study (data not shown).

Poor health behaviours are another potential mechanism linking disability discrimination and poorer well-being. For example, perceived disability discrimination has been linked with worse sleep quality in the HRS, with psychological distress acting as a full mediator of this association.^45^ It is possible that disabled people could engage in negative health behaviours as a means of coping with the psychological impact of discrimination. In a study of 304 individuals with disability, perceived disability discrimination was positively associated with illicit drug use.^46^ Eating may offer a source of comfort in the face of discrimination.^47^ A US study of over 5000 individuals observed a link between physical disability discrimination and overeating.^48^

Another possibility is that perceived disability discrimination and well-being are linked through disturbed stress-related biological processes. In line with the theory of allostatic load, perceived chronic discrimination causing frequent activation of the stress response system, could over time result in disturbances across multiple biological systems.^49^ Systematic reviews and meta-analyses, which have predominately focused on racism, suggest that discrimination is linked with heightened cardiovascular stress reactivity,^14 50^ while race^50–52^ and weight discrimination^53^ have been linked with alterations in cortisol. To our knowledge, no study has investigated associations between perceived disability discrimination and changes in cardiovascular or neuroendocrine activity. In the HRS cohort, perceived disability discrimination was linked with raised C reactive protein levels cross-sectionally.^54^ Heightened inflammation is thought to be predictive of poorer mental well-being,^55^ offering a plausible pathway between perceived disability discrimination and later psychological distress and poorer mental functioning seen in the present study. Further work is required to confirm this assertion, particularly as the HRS analysis was not limited to those with a confirmed disability.

Our study had several strengths. The use of the UKHLS cohort allowed us to examine well-being over a 4-year period across a wide age range (17–96 years), while adjusting statistically for factors that could confound associations. The discrimination measure took into account several kinds of discriminatory behaviour and included multiple settings where perceived disability discrimination could be encountered.

However, the study was not without limitations. We lost a considerable number of participants at follow-up, and although the cross-sectional findings did not differ between those who provided data at both waves and those lost to follow-up (sensitivity analysis 1), we cannot be sure that selection bias due to low retention did not impact our findings longitudinally due to the extent of missing data. Indeed, in imputed analyses (sensitivity analysis 3) the association between disability discrimination and well-being held for SF-12 mental functioning but not for psychological distress. Our findings are based on perceptions of disability discrimination rather than objective encounters with disability discrimination. It is possible that perceiving oneself as a target for discrimination and objective encounters with discrimination could have differing consequences for well-being. Indeed, earlier work in a sample with significant health limitations indicates that individuals with poorer mental well-being may be more likely to perceive stigma.^56^ Future studies assessing reciprocal prospective associations between perceived disability discrimination and well-being could help to clarify this issue. Our discrimination measure was based on self-reports of experiences during the past 12 months and was therefore subject to recall bias. Furthermore, this measure was not specific to disability discrimination. The fact that participants were able to attribute multiple reasons for their experience of discrimination, could have helped avoid priming or bias. Other tools specifically designed to assess disability discrimination could have garnered different results. Our sample was ethnically diverse, and we took ethnicity into account in our models. Although disability discrimination was the most commonly reported form of discrimination in this sample, perceived discrimination on the basis of ethnicity may also have been relevant for this sample. Further work is required to understand how disability discrimination interacts with ethnicity discrimination, as well as other types of discrimination to influence well-being. Disability discrimination was only assessed at one point in time, meaning our measure does not necessarily reflect pervasive discrimination. However, other work in UKHLS suggests that perceived disability discrimination is still frequently reported at later stages of data collection.^57^ Future research is required to determine whether perceptions of disability discrimination are persistent or alter over time. We operationalised perceived discrimination as a simple binary variable and had no information on the frequency of encounters with discrimination over time. Therefore, the potential dose-response relationship between the frequency of discrimination and well-being remains to be elucidated. Our study included participants with physical, cognitive and sensory disabilities. However, our sample is unlikely to have captured those with severe cognitive impairments due to the demands of survey participation. For a large proportion (37%), their disability type was unknown and classified as ‘other’, limiting our understanding. While, no one with a mental health-related disability was included in the physical, cognitive and sensory disabilities categories, we cannot be certain that the ‘other’ category did not include participants with mental health-related impairments.

Overall, our study adds to the literature by demonstrating prospective associations between perceived disability discrimination and well-being outcomes. These findings emphasise the need to reduce the prevalence of disability discrimination, with the benefit of promoting equality as well as possible advantages for well-being too. Although complete elimination of disability discrimination is likely to be difficult, recognition of disability discrimination as an issue is the first step in preventing its occurrence. Addressing this could involve raising awareness through the use of campaigns. The Public Sector Equality Duty in the UK requires public bodies to have due regard to the need to eliminate discrimination and this awareness raising should begin early in life.^7^ However, it is estimated that <40% of English primary schools have a disability equality scheme in place, with race and gender equality more often prioritised over disability equality.^58^ Therefore, further effort on this issue is required,^7^ particularly as disability discrimination is perceived to be more widespread than gender discrimination in Europe.^8^

As well as macro-level awareness raising, on an individual basis the negative impact of perceived disability discrimination on well-being may be buffered through the use of social support. In two cross-sectional studies of US adults with varied disability diagnoses, those with more friends reported greater life satisfaction and these friendships attenuated the link between functional impairment and poorer quality of life.^59^ In an Israeli study, perceived disability discrimination and poorer life satisfaction were only linked in those with low and moderate levels of social support, with no association in those with greater levels of support.^60^ Further research on disability discrimination is necessary to develop awareness campaigns and to appropriately target individual-level interventions.

## Supplementary Material

Reviewer comments

Author's manuscript

## References

[1] Legislation.gov.uk The equality act, 2010 Available: https://www.legislation.gov.uk/ukpga/2010/15/contents

[2] World Health Organisation World report on disability 2011. World Health Organisation, 2011.

[3] Leonardi M, Bickenbach J, Ustun TB, et al The definition of disability: what is in a name? The Lancet 2006;368:1219–21. 10.1016/S0140-6736(06)69498-1 17027711

[4] Department for Work and Pensions Family resources survey 2017-18, 2019 Available: https://assets.publishing.service.gov.uk/government/uploads/system/uploads/attachment_data/file/790000/family-resources-survey-2017-18.pdf

[5] Equality and Human Rights Commission Being disabled in Britain: a journey less equal, 2017.

[6] Office for National Statistics. Life opportunities survey: wave 3 results., 2015 Available: https://www.gov.uk/government/statistics/life-opportunities-survey-wave-3-results

[7] Equality and Human Rights Commission Hidden in plain sight: inquiry into disability-related harassment., 2011 Available: https://www.equalityhumanrights.com/en/publication-download/hidden-plain-sight-inquiry-disability-related-harassment [Accessed 28 Mar 2019].

[8] European Union Discrimination in the EU in 2015, 2015.

[9] Sykes W, Groom C, Desai P Disability-related harassment: the role of public bodies a qualitative research report. Equality and Human Rights Commission: Manchester:, 2011.

[10] Office for National Statistics Crime survey for England and Wales 2014-2015, 2016.

[11] Scottish Government Report of Independent Advisory Group on Hate Crime, Prejudice and Community Cohesion’, 2016 Available: https://www.gov.scot/publications/report-independent-advisory-group-hate-crime-prejudice-community-cohesion/

[12] Corcoran H, Lader D, Smith K Hate crime, England and Wales 2014 to 2015, 2015.

[13] Coleman N, Sykes W, Groom C Barriers to employment and unfair treatment at work: a quantitative analysis of disabled people’s experiences. Equality and Human Rights Commission, 2013.

[14] Pascoe EA, Smart Richman L Perceived discrimination and health: a meta-analytic review. Psychol Bull 2009;135:531–54. 10.1037/a0016059 19586161 PMC2747726

[15] Goto JB, Couto PFM, Bastos JL [Systematic review of epidemiological studies on interpersonal discrimination and mental health]. Cad Saude Publica 2013;29:445–59. 10.1590/s0102-311x2013000300004 23532281

[16] Schmitt MT, Branscombe NR, Postmes T, et al The consequences of perceived discrimination for psychological well-being: a meta-analytic review. Psychol Bull 2014;140:921–48. 10.1037/a0035754 24547896

[17] Ali A, King M, Strydom A, et al Self-Reported stigma and symptoms of anxiety and depression in people with intellectual disabilities: findings from a cross sectional study in England. J Affect Disord 2015;187:224–31. 10.1016/j.jad.2015.07.046 26342918

[18] Temple JB, Kelaher M Is disability exclusion associated with psychological distress? Australian evidence from a national cross-sectional survey. BMJ Open 2018;8:e020829 10.1136/bmjopen-2017-020829 PMC5988124 29794096

[19] Krnjacki L, Priest N, Aitken Z, et al Disability-based discrimination and health: findings from an Australian-based population study. Aust N Z J Public Health 2018;42:172–4. 10.1111/1753-6405.12735 29168323

[20] Wamala S, Boström G, Nyqvist K Perceived discrimination and psychological distress in Sweden. Br J Psychiatry 2007;190:75–6. 10.1192/bjp.bp.105.021188 17197660

[21] Daley A, Phipps S, Branscombe NR The social complexities of disability: discrimination, belonging and life satisfaction among Canadian youth. SSM Popul Health 2018;5:55–63. 10.1016/j.ssmph.2018.05.003 29892696 PMC5993176

[22] Kim S A study of the impact of discrimination experience on life satisfaction in Korean women with severe disabilities. World Acad Sci Eng Technol Int J Soc Behav Educ Econ Bus Ind Eng 2015;9:3205–9.

[23] Kagan M, Itzick M, Tal-Katz P, Demographic T-KP Demographic, psychosocial, and health- and disability-related factors associated with psychological distress among people with physical disabilities. Rehabil Psychol 2018;63:392–9. 10.1037/rep0000206 30047749

[24] Du Mont J, Forte T Perceived discrimination and self-rated health in Canada: an exploratory study. BMC Public Health 2016;16:742 10.1186/s12889-016-3344-y 27502071 PMC4977718

[25] Alvarez-Galvez J, Salvador-Carulla L Perceived discrimination and self-rated health in Europe: evidence from the European social survey (2010). PLoS One 2013;8:e74252 10.1371/journal.pone.0074252 24040216 PMC3764018

[26] Emerson E Self-Reported exposure to disablism is associated with poorer self-reported health and well-being among adults with intellectual disabilities in England: a cross-sectional survey. Public Health 2010;124:682–9. 10.1016/j.puhe.2010.08.020 21035153

[27] Sutin AR, Stephan Y, Carretta H, et al Perceived discrimination and physical, cognitive, and emotional health in older adulthood. Am J Geriatr Psychiatry 2015;23:171–9. 10.1016/j.jagp.2014.03.007 24745563 PMC4170050

[28] University of Essex Institute for Social and Economic Research, NatCen Social Research, Kantar Public : Understanding Society: Waves 1-7, 2009-2016 and Harmonised BHPS: Waves 1-18, 1991-2009. 9th Edition UK: data service, 2017.

[29] Knies G Understanding society: The UK household longitudinal study, Waves 1–7 (User Guide). Institute for Social and Economic Research: University of Essex: : Colchester, 2017.

[30] McFall S, Nandi A, Platt L Understanding Society: UK household longitudinal study: user guide to ethnicity and immigration research. Colchester: University of Essex, 2017.

[31] Goldberg D Williams P. A user’s guide to the General Health Questionnaire. Basingstoke: NFER-Nelson, 1988.

[32] Ware J, Kosinski M, Keller SD A 12-Item short-form health survey: construction of scales and preliminary tests of reliability and validity. Med Care 1996;34:220–33. 10.1097/00005650-199603000-00003 8628042

[33] Ware JE How to score version 2 of the SF-12 health survey (with a supplement documenting version 1). 1st Lincoln, RI: Health Assessment Lab, 2002.

[34] Steptoe A, Jackson SE The life skills of older Americans: association with economic, psychological, social, and health outcomes. Sci Rep 2018;8:9669 10.1038/s41598-018-27909-w 29977019 PMC6033934

[35] DeSalvo KB, Bloser N, Reynolds K, et al Mortality prediction with a single General self-rated health question. A meta-analysis. J Gen Intern Med 2006;21:267–75. 10.1111/j.1525-1497.2005.00291.x 16336622 PMC1828094

[36] Hackett RA, Steptoe A, Jackson SE Sex discrimination and mental health in women: a prospective analysis. Health Psychol 2019;38:1014–24. 10.1037/hea0000796 31497985

[37] World Health Organisation Depression and Other Common Mental Disorders: Global Health Estimates. Geneva: World Health Organisation, 2017.

[38] Wamala S, Merlo J, Boström G, et al Perceived discrimination, socioeconomic disadvantage and refraining from seeking medical treatment in Sweden. J Epidemiol Community Health 2007;61:409–15. 10.1136/jech.2006.049999 17435207 PMC2465685

[39] Sareen J, Afifi TO, McMillan KA, et al Relationship between household income and mental disorders: findings from a population-based longitudinal study. Arch Gen Psychiatry 2011;68:419–27. 10.1001/archgenpsychiatry.2011.15 21464366

[40] OECD What are equivalence scales? 2005.

[41] Alvarez A, Faccioli J, Guinzbourg M, et al Endocrine and inflammatory profiles in type 2 diabetic patients with and without major depressive disorder. BMC Res Notes 2013;6:61 10.1186/1756-0500-6-61 23410093 PMC3599430

[42] Rogers SE, Thrasher AD, Miao Y, et al Discrimination in healthcare settings is associated with disability in older adults: health and retirement study, 2008-2012. J Gen Intern Med 2015;30:1413–20. 10.1007/s11606-015-3233-6 25773918 PMC4579241

[43] Moscoso-Porras MG, Alvarado GF Association between perceived discrimination and healthcare-seeking behavior in people with a disability. Disabil Health J 2018;11:93–8. 10.1016/j.dhjo.2017.04.002 28420592

[44] Ali A, Scior K, Ratti V, et al Discrimination and other barriers to accessing health care: perspectives of patients with mild and moderate intellectual disability and their carers. PLoS One 2013;8:e70855 10.1371/journal.pone.0070855 23951026 PMC3741324

[45] Vaghela P, Sutin AR Discrimination and sleep quality among older us adults: the mediating role of psychological distress. Sleep Health 2016;2:100–8. 10.1016/j.sleh.2016.02.003 28923251

[46] Li Li DM Disability and illicit drug use: an application of labeling theory. Deviant Behav 2001;22:1–21.

[47] Wansink B, Cheney MM, Chan N Exploring comfort food preferences across age and gender. Physiol Behav 2003;79:739–47. 10.1016/S0031-9384(03)00203-8 12954417

[48] Sutin A, Robinson E, Daly M, et al Weight discrimination and unhealthy eating-related behaviors. Appetite 2016;102:83–9. 10.1016/j.appet.2016.02.016 26877216 PMC4866877

[49] McEwen BS, Stress MBS Stress, adaptation, and disease. allostasis and allostatic load. Ann N Y Acad Sci 1998;840:33–44. 10.1111/j.1749-6632.1998.tb09546.x 9629234

[50] Lockwood KG, Marsland AL, Matthews KA, et al Perceived discrimination and cardiovascular health disparities: a multisystem review and health neuroscience perspective. Ann N Y Acad Sci 2018;1428:170–207. 10.1111/nyas.13939 30088665

[51] Busse D, Yim IS, Campos B, et al Discrimination and the HPA axis: current evidence and future directions. J Behav Med 2017;40:539–52. 10.1007/s10865-017-9830-6 28155003

[52] Korous KM, Causadias JM, Casper DM Racial discrimination and cortisol output: a meta-analysis. Soc Sci Med 2017;193:90–100. 10.1016/j.socscimed.2017.09.042 29028560

[53] Jackson SE, Kirschbaum C, Steptoe A Perceived weight discrimination and chronic biochemical stress: a population-based study using cortisol in scalp hair. Obesity 2016;24:2515–21. 10.1002/oby.21657 27740706 PMC5132135

[54] Sutin AR, Stephan Y, Luchetti M, et al Perceived weight discrimination and C-reactive protein. Obesity 2014;22:1959–61. 10.1002/oby.20789 24828961

[55] Slavich GM, Irwin MR From stress to inflammation and major depressive disorder: a social signal transduction theory of depression. Psychol Bull 2014;140:774–815. 10.1037/a0035302 24417575 PMC4006295

[56] Alonso J, Buron A, Bruffaerts R, et al Association of perceived stigma and mood and anxiety disorders: results from the world mental health surveys. Acta Psychiatr Scand 2008;118:305–14. 10.1111/j.1600-0447.2008.01241.x 18754833 PMC3040096

[57] Emerson E, Krnjacki L, Llewellyn G, et al Perceptions of safety and exposure to violence in public places among working age adults with disabilities or long-term health conditions in the UK: cross sectional study. Public Health 2016;135:91–6. 10.1016/j.puhe.2015.10.036 26976491

[58] Beckett AE, Buckner L Promoting positive attitudes towards disabled people: definition of, rationale and prospects for anti-disablist education. Br J Sociol Educ 2012;33:873–91. 10.1080/01425692.2012.692046

[59] Silverman AM, Molton IR, Smith AE, et al Solace in solidarity: disability friendship networks buffer well-being. Rehabil Psychol 2017;62:525–33. 10.1037/rep0000128 28394146

[60] Itzick M, Kagan M, Tal-Katz P Perceived social support as a moderator between perceived discrimination and subjective well-being among people with physical disabilities in Israel. Disabil Rehabil 2018;40:2208–16. 10.1080/09638288.2017.1331380 28549403

