# Supplementary material for: Disability discrimination and well-being in the United Kingdom: a prospective cohort study
Source: BMJ Open. 2020 Mar 12;10(3):e035714. doi: 10.1136/bmjopen-2019-035714 (PMC7069317; doi:10.1136/bmjopen-2019-035714)
Supplement: Supplementary data [file bmjopen-2019-035714supp001.pdf]

Supplementary table 1: Perceived disability discrimination types and settings by type of disability

**Types of discrimination**	**Physical**	**Sensory**	**Cognitive**	**Other**	***p* value**
Felt unsafe at someplace	12 (85.7%)	9 (90%)	12 (92.3%)	60 (84.5%)	0.874
Avoided at someplace	8 (53.3%)	6 (75%)	9 (75%)	52 (69.3%)	0.336
Felt insulted at someplace	3 (25%)	1 (12.5%)	1 (10%)	15 (27.3%)	0.572
Attacked at someplace	0 (0%)	0 (0%)	0(0%)	3(4.4%)	0.621
**Settings**					
School/work	2 (12.5%)	1 (9.1%)	0 (0%)	12 (15.8%)	0.424
Public transport	10 (62.5%)	3 (27.3%)	6 (42.9%)	41 (53.9%)	0.266
Bus or train stations	5 (31.3%)	2 (18.2%)	6 (42.9%)	34 (44.7%)	0.328
Taxis	4 (25%)	0 (0%)	1 (7.1%)	9 (11.8%)	0.223
Public buildings	8 (50%)	7 (63.6%)	7 (50%)	48 (63.2%)	0.654
On the street	12 (75%)	8 (72.7%)	9 (64.3%)	62 (81.6%)	0.504
At home	3 (18.8%)	3 (27.3%)	4 (28.6%)	20 (26.3%)	0.920

Data are presented as numbers (% yes)

Supplementary table 2: Prospective associations between perceived disability discrimination and wellbeing outcomes (imputed analysis)

	**Psychological distress**[a]	**SF-12 mental**[b]	**Life satisfaction**[c]	**Fair/poor self-rated health**[d]
	B (95% CI)	*B* (95% CI)	*B* (95% CI)	*B* (95% CI)
**Wave 5**				
No perceived	Reference	Reference	Reference	1 (Reference)
discrimination	0.22 [-3.21; 3.64]			1.38 [0.65; 2.93]
Perceived discrimination		-4.55 [-8.75; -0.34]*	-0.49 [-1.10; 0.12]	

All analyses are adjusted for age, sex, household income, education, ethnicity and disability type. Prospective analyses are additionally adjusted for baseline wellbeing status/score.

[a]= *n*= 454 for the no perceived discrimination group; *n*= 82 for the perceived discrimination group; [b]= *n*= 742 for the no perceived discrimination group; *n*= 117 for the perceived discrimination group; [c]= *n*= 454 for the no perceived discrimination group; *n*= 84 for the perceived discrimination group; [d]= *n*= 754 for the no perceived discrimination group; *n*= 117 for the perceived discrimination group

*p<0.05, **p<0.01, ***p <0.001

CI = confidence interval, OR = odds ratio

Possible scores on the psychological distress scale range from 0-12, SF-12 mental component scale range from 0-100 and the life satisfaction scale scores range from 0-7.
