# Supplementary material for: Disability discrimination and well-being in the United Kingdom: a prospective cohort study
Source: BMJ Open. 2020 Mar 12;10(3):e035714. doi: 10.1136/bmjopen-2019-035714 (PMC7069317; doi:10.1136/bmjopen-2019-035714)
Supplement: Author's manuscript [file bmjopen-2019-035714.draft_revisions.pdf]

BMJ Open is committed to open peer review. As part of this commitment we make the peer review history of every article we publish publicly available.

When an article is published we post the peer reviewers' comments and the authors' responses online. We also post the versions of the paper that were used during peer review. These are the versions that the peer review comments apply to.

The versions of the paper that follow are the versions that were submitted during the peer review process. They are not the versions of record or the final published versions. They should not be cited or distributed as the published version of this manuscript.

BMJ Open is an open access journal and the full, final, typeset and author-corrected version of record of the manuscript is available on our site with no access controls, subscription charges or pay-per-view fees (http://bmjopen.bmj.com).

If you have any questions on BMJ Open's open peer review process please email info.bmjopen@bmj.com

BMJ Open

Disability discrimination and mental health: A prospective analysis

Journal:	*BMJ Open*
Manuscript ID	bmjopen-2019-035714
Article Type:	Original research
Date Submitted by the Author:	13-Nov-2019

Complete List of Authors:	Hackett, Ruth; University College London, Behavioural Science and Health; King's College London, Health Psychology Section<br>Lang, Raymond; University College London, Leonard Cheshire Research Centre, Epidemiology and Public Health<br>Steptoe, Andrew; University College London, Behavioural Science and Health<br>Jackson, Sarah ; University College London, Behavioural Science and Health

Keywords:	EPIDEMIOLOGY, MENTAL HEALTH, PUBLIC HEALTH

SCHOLARONE™
Manuscripts

I, the Submitting Author has the right to grant and does grant on behalf of all authors of the Work (as defined in the below author licence), an exclusive licence and/or a non-exclusive licence for contributions from authors who are: i) UK Crown employees; ii) where BMJ has agreed a CC-BY licence shall apply, and/or iii) in accordance with the terms applicable for US Federal Government officers or employees acting as part of their official duties; on a worldwide, perpetual, irrevocable, royalty-free basis to BMJ Publishing Group Ltd ("BMJ") its licensees and where the relevant Journal is co-owned by BMJ to the co-owners of the Journal, to publish the Work in this journal and any other BMJ products and to exploit all rights, as set out in our licence.

The Submitting Author accepts and understands that any supply made under these terms is made by BMJ to the Submitting Author unless you are acting as an employee on behalf of your employer or a postgraduate student of an affiliated institution which is paying any applicable article publishing charge ("APC") for Open Access articles. Where the Submitting Author wishes to make the Work available on an Open Access basis (and intends to pay the relevant APC), the terms of reuse of such Open Access shall be governed by a Creative Commons licence – details of these licences and which Creative Commons licence will apply to this Work are set out in our licence referred to above.

Other than as permitted in any relevant BMJ Author's Self Archiving Policies, I confirm this Work has not been accepted for publication elsewhere, is not being considered for publication elsewhere and does not duplicate material already published. I confirm all authors consent to publication of this Work and authorise the granting of this licence.

For peer review only

Disability discrimination and mental health: A prospective analysis

Ruth A. Hackett[1,2*], Andrew Steptoe[1], Raymond P. Lang[3], and Sarah E. Jackson[1]

[1] Department of Behavioural Science and Health, University College London, London, UK

[2] Health Psychology Section, Department of Psychology, Institute of Psychiatry, Psychology and Neuroscience, Guy's Hospital, King's College London, London, UK

[3] Leonard Cheshire Research Centre, Department of Epidemiology and Public Health, University College London, London, UK

***Corresponding author:** Ruth A. Hackett, PhD. Department of Behavioural Science and Health, University College London and Health Psychology Section, Department of Psychology, Institute of Psychiatry, Psychology and Neuroscience, Guy's Hospital, King's College London, London, UK

Email: ruth.hackett.09@ucl.ac.uk ORCID: https://orcid.org/0000-0002-5428-2950

Co-author emails: a.steptoe@ucl.ac.uk r.lang@ucl.ac.uk s.e.jackson@ucl.ac.uk

Word count: 3981

Number of tables: 5

For peer review only - http://bmjopen.bmj.com/site/about/guidelines.xhtml

Abstract

Objectives: Disability discrimination is linked with poorer mental health cross-sectionally. The aim of this study was to explore prospective associations between disability discrimination and mental wellbeing.

Design: Prospective cohort study

Setting: The United Kingdom Household Longitudinal Study

Participants: Data were from 871 individuals with a self-reported physical, cognitive or sensory disability.

Primary outcome measures: Depression was assessed in 2009/10. Psychological distress, mental functioning, life satisfaction and self-rated health were assessed in 2009/10 and 2013/14.

Results: Data were analysed using linear and logistic regression with adjustment for age, sex, household income, education, ethnicity and impairment category. Perceived disability discrimination was reported by 117 (13.4%) participants. Cross-sectionally, discrimination was associated with depression (Odds ratio (OR) = 5.40, 95% Confidence Interval (CI) 3.25; 8.97) fair/poor self-rated health (OR=2.05; 95% CI 1.19; 3.51), greater psychological distress (*B* = 3.28, 95% CI 2.41; 4.14), poorer mental functioning (*B* = -7.35; 95% CI -9.70; -5.02) and life satisfaction (*B* = -1.27, 95% CI -1.66; -0.87). Prospectively, discrimination was associated with increased psychological distress (*B* = 2.88, 95% CI 1.39; 4.36) and poorer mental functioning (*B* = -5.12; 95% CI -8.91; -1.34), adjusting for baseline scores.

Conclusions: Perceived disability-related discrimination is linked with poorer mental wellbeing. These findings underscore the need for interventions to combat disability discrimination.

Key words: disability, discrimination; prejudice; stigma; mental health; wellbeing

For peer review only - http://bmjopen.bmj.com/site/about/guidelines.xhtml

Article Summary

- The use of the prospective United Kingdom Household Longitudinal Study allowed use to examine changes in mental wellbeing over a 4 year period.
- The disability discrimination measure took into account several kinds of discriminatory behaviour and included multiple settings where perceived disability discrimination could be encountered.
- Our findings are based on perceptions of disability discrimination rather than objective encounters with disability discrimination.
- Disability discrimination was only assessed at one point in time, meaning our measure does not necessarily reflect pervasive discrimination.

Funding: This work was supported by the Economic and Social Research Council (https://esrc.ukri.org/), grant number ES/R005990/1. The funders had no role in study design, data collection and analysis, decision to publish, or preparation of the manuscript.

Competing interests: The authors declare that they have no conflict of interest.

Data sharing: The UKHLS datasets analysed during the current study are freely available in the UK Data Service repository https://ukdataservice.ac.uk/

For peer review only - http://bmjopen.bmj.com/site/about/guidelines.xhtml

Introduction

"Disability" is an umbrella term for long-term impairments, activity limitations and participation restrictions, experienced by an individual with a health condition in interaction with their environment[1–3]. In the UK, the prevalence of disability is rising, with 21% of the population reporting a disability in 2017-2018, an increase from 18% in 2007-2008[4]. The majority of disabled people report experiencing difficulties in everyday life. For example, disabled people in Britain have lower educational attainment, are less likely to be employed and are more likely to live in poverty than non-disabled adults[5], with knock on effects regarding access to health and other services[6].

These practical difficulties may be aggravated by discrimination. Disability discrimination has been defined as unwanted, exploitative or abusive conduct against disabled people which violates their dignity and security or creates an intimidating or offensive environment[7]. Although disability is a protected characteristic under equality legislation[1], disability discrimination is perceived to be common. In a 2015 population survey of 27,718 adults from 26 European countries, 50% of participants reported disability discrimination to be widespread, a 4% increase from 2012 data[8].

Disability discrimination can happen in a variety of settings including on the street, in the workplace and in public venues such as shops or pubs[9]. Recent British data suggests that disabled people are more likely than non-disabled people to report feeling unsafe when walking alone and to worry about physical attack and theft [10,11]. Disability is the second most common motivator for hate crime incidents, after race in England and Wales[12]. In the workplace, several studies suggest that a greater proportion of disabled than non-disabled individuals report experiences of discrimination [7,13].

A growing body of research has investigated discrimination as a determinant of mental wellbeing[14–16]. Meta-analyses have linked perceived discrimination with depression and

For peer review only - http://bmjopen.bmj.com/site/about/guidelines.xhtml

psychological distress and with poorer life satisfaction and self-rated health[14–16]. However, disability discrimination was not assessed in two of these meta-analyses and the majority of previous working has focused on racism [14,15]. In the most recent pooled analysis of 328 studies, physical illness discrimination and disability discrimination were assessed as a combined category[16], with associations between discrimination and greater psychological distress and lower self-esteem detected.

Several cross-sectional studies have assessed perceived disability discrimination alone in relation to mental wellbeing outcomes. One study of 229 individuals with an intellectual disability in England found that self-reported stigma was associated with a greater number of depression and anxiety symptoms[17]. Two analyses of the Australian Survey of Disability, Ageing and Carers (n=9655 and n=6183, respectively) linked disability discrimination with greater psychological distress[18,19]. This association was similarly observed in a Swedish general population survey[20]. Research has also linked perceived disability discrimination with lower life satisfaction in Canadian adolescents[21], Korean women with severe disabilities[22] and Israeli nationals with physical disabilities[23]. Perceived disability discrimination has been associated with poorer self-rated health cross-sectionally in three studies[19,24,25] including a general population analysis of 52,458 individuals, from the European Social Survey[25].

Cross-sectional correlations are difficult to interpret: perception of discrimination may result in emotional distress, but it is also possible that emotional distress leads to alterations in how people interpret social interactions with others. To date, only one study has assessed prospective associations between perceived disability discrimination and mental health and wellbeing outcomes[26]. In an analysis of older adults (≥50 years) participating in the US-based Health and Retirement Study (HRS), perceived disability discrimination was associated with poorer life satisfaction, self-rated health, and greater loneliness over four-year follow-up.

For peer review only - http://bmjopen.bmj.com/site/about/guidelines.xhtml

Overall, previous research has been dominated by cross-sectional studies, precluding the assessment of the temporal relationship between perceived disability discrimination and mental wellbeing outcomes. No longitudinal studies have compared people with a disability who do or do not report discrimination and wellbeing outcomes. To address these limitations, this study aimed to investigate cross-sectional and prospective associations between perceived disability discrimination and mental wellbeing in a UK population cohort.

Methods

Study population

This study uses data from Understanding Society: The UK Household Longitudinal Study (UKHLS)[27]. Data collection began in 2009/10 (wave 1) with follow-ups annually. This study uses data from waves 1 (2009/10) and 5 (2013/14). The UKHLS comprises of a representative sample of the UK population, in addition to an ethnic minority boost sample[28,29]. Our data come from the 'extra 5 minutes sample' of over 8,000 participants who had an additional 5 minutes of questions on issues pertinent to ethnicity research including discrimination. This sample comprises of mostly ethnic minorities (*n* = 6722) along with a comparison group of white participants (*n* = 1428)[28]. We restricted our sample to those who responded to the disability discrimination questions (*n* = 4788) with a self-reported disability (*n* = 871). At wave 5 there was loss to follow-up (n=431), leaving a follow-up sample size of 440 participants. Our definition of disability did not include mental health-related impairments. Ethical approval for UKHLS was obtained from the University of Essex Ethics Committee. All participants provided fully informed consent.

Perceived disability discrimination

For peer review only - http://bmjopen.bmj.com/site/about/guidelines.xhtml

To assess discrimination, participants were asked whether in the past 12 months they (a) felt unsafe, (b) avoided going to or being in, (c) had been insulted, called names, threatened or shouted at, or (d) had been physically attacked, in seven different settings: 1) At school/college/work, 2) On public transport, 3) At or around bus or train stations, 4) In a taxi, 5) Public buildings such as shopping centres or pubs, 6) Outside on the street, in parks or other public places, or 7) At home. If they answered yes, a follow-up question asked them to choose a reason from a list of categories including disability, sex and ethnicity among others. It was possible to choose multiple settings and attributions for the perceived discrimination. Those who attributed any experience of discrimination to disability are treated as cases of perceived disability discrimination.

Outcome variables

Self-reported doctor-diagnosed clinical depression was measured at wave 1 (2009/10) with responses coded as yes/no. Depression was not analysed longitudinally due to a lack of incident cases. All other outcomes were assessed at waves 1 (2009/10) and 5 (2013/14). Psychological distress was measured using the General Health Questionnaire (GHQ)-12[30], which involved ratings of 12 statements including whether the individual had "*Been able to enjoy your normal day to day activities*" or whether they "*Felt constantly under strain*" with response options of 0="no" and 1="yes". Total scores range from 0 (least distressed) to 12 (most distressed). The 12-item short-form health survey (SF-12) mental component summary score was used to measure limitations caused by emotional, mental health and social functioning issues[31]. Items included ratings of feelings experienced over the past 4 weeks such as "*Have you felt downhearted or blue*?" or *"Accomplished less than you would like"*. Overall scores were derived using standard methods ranging from 0 (low functioning) to 100 (high functioning) [32]. Life satisfaction was assessed using one item asking participants how satisfied they were

For peer review only - http://bmjopen.bmj.com/site/about/guidelines.xhtml

with their "life overall", with scores ranging from 1 (completely dissatisfied) to 7 (completely satisfied). Self-rated health was assessed using a single item: "*Would you say your health is…poor/fair/good/very good/excellent?"* In keeping with previous investigations [33,34] self-rated health was dichotomised with 0 being "good/very good/excellent" and 1 meaning "poor/fair".

Covariates

A number of covariates (assessed at wave 1) that are likely relevant to perceived disability discrimination and mental health were included in our analyses. Age in years was entered as a continuous variable. Sex was included as a binary variable (male/female). Equivalised monthly household income was calculated by dividing total household net income by the modified Organization for Economic Cooperation and Development (OECD) equivalence scale to adjust for the effects of household size and composition[35]. Education was included as a 3-level variable: 1 "university degree", 2 "high school qualification" and 3 "no qualification". As our sample was ethnically diverse, we included ethnicity as a 4-level variable with 1 being "white" including those of white British, white Irish and any other white background, 2 being "south Asian" including Indian, Pakistani and Bangladeshi individuals, 3 being "black" including black African and black Caribbean participants and 4 being "other" including individuals from Chinese and mixed backgrounds. There were 4 categories of impairment measured in the study: "physical" disability which included difficulties with manual dexterity and mobility; "cognitive" disability including problems with memory or the ability to concentrate, learn and understand; "sensory" disability including hearing (apart from using a standard hearing aid) and sight impairments (apart from wearing standard glasses) and "other" which encompassed reports of unspecified disability not captured in the other categories.

For peer review only - http://bmjopen.bmj.com/site/about/guidelines.xhtml

Statistical analyses

We compared the characteristics of those who did and those who did not report disability discrimination at wave 1 using Chi-squared tests for categorical variables and independent samples t-tests for continuous variables. Associations between perceived disability discrimination and the various wellbeing measures were assessed using linear regression for continuous outcomes and logistic regression for categorical outcomes. For cross-sectional analyses, depression, psychological distress (GHQ-12), SF-12 mental component score, life satisfaction and self-rated health at wave 1 (2009/10) were the outcome variables. For prospective analyses, psychological distress (GHQ-12), SF-12 mental component score, life satisfaction and self-rated health at wave 5 (2013/14) were the outcomes. Age, sex, household income, education, ethnicity and disability type at wave 1 were controlled for in all analyses. Baseline (wave 1) scores/status on the relevant wellbeing variable was included as an additional covariate in prospective analyses. We tested for interactions between perceived disability discrimination and age, sex, income, education, ethnicity or impairment type on wellbeing at wave 5. No significant effects were detected. Thus, interaction terms were not included in our models. Results from linear regression analyses are presented as unstandardized B and 95% confidence intervals (95% CI). Results from logistic regression analyses are presented as odds ratios (ORs) and 95% CI. All analyses were conducted using SPSS v.24.

Sensitivity analyses

We carried out two sensitivity analyses. In our first, we assessed whether those who were lost to follow-up (n = 431) differed from those who provided data at both waves (n = 440). We tested whether this impacted the results by conducting the cross-sectional analyses (wave 1) including only those who provided follow-up data at wave 5. In our second sensitivity analysis, we assessed whether a certain type of discriminatory behaviour contributing to the measure of

For peer review only - http://bmjopen.bmj.com/site/about/guidelines.xhtml

perceived disability discrimination was driving the results. We tested this cross-sectionally and prospectively removing each type of discriminatory behaviour in turn.

Results

A total of 871 participants were included in the study and of these 117 (13.4%) reported perceived disability discrimination. Of the categories of discrimination assessed, the most commonly reported was feeing unsafe (86.1%; 95% CI 79.48-92.74), followed by avoiding somewhere (72.8%; 95% CI 64.08-81.55), being insulted (23.5%; 95% CI 14.33-32.73) and being physically attacked (2.8%; 95% CI 0.04-5.98). The most common settings in which disability discrimination was reported were on the street (77.8%; 95% CI 70.13-85.42), in public buildings such as shops or pubs (59.8%; 95% CI 50.81-68.84), on public transport (51.3%; 95% CI 42.09-60.47) and at or around bus or train stations (40.2%; 95% CI 31.16-49.19). A quarter of participants reported experiencing disability discrimination at home (25%; 95% CI 17.61-33.67). Perceived disability discrimination was less frequently reported in school or workplace settings (12.8%; 95% CI 6.67-18.97) or in taxis (12%; 95% CI 6-17.93). The prevalence of the various types of perceived disability discrimination and the settings in which the discrimination occurred for different types of disability can be found in Supplementary Table 1. There were no statistically significant differences between people with different types of disability in discrimination type or discrimination setting.

The baseline characteristics of the sample are displayed in Table 1. The group who reported disability discrimination were younger on average (48.29 ± 14.89 years) than those who did not report discrimination (53.42 ± 16.56 years). They were more likely to be white (27.4% vs. 20.3%) and to be better educated than those who did not report discrimination, with a greater proportion holding university degrees (28.2% vs 22.7%). Physical disability was most

For peer review only - http://bmjopen.bmj.com/site/about/guidelines.xhtml

common in those who did not perceive discrimination (46.6%), whereas other unspecified disabilities (65%) were most frequently reported by those who perceived discrimination.

Cross-sectional associations between perceived disability discrimination and wellbeing

Our findings suggest that individuals who perceived disability discrimination were significantly more likely to report a diagnosis of clinical depression (OR=5.40; 95% CI 3.25; 8.97, p <0.001) and were more likely to rate their health as fair/poor (OR=2.05; 95% CI 1.19; 3.51, p =0.009) than those who did not perceive disability discrimination, independent of covariates (first panel Table 2). Those who reported discrimination also had significantly higher levels of psychological distress (B = 3.28, 95% CI 2.41; 4.14, p <0.001), poorer mental functioning on the SF-12 (B = -7.35; 95% CI -9.70; -5.02, p <0.001) and lower life satisfaction (B = -1.27, 95% CI -1.66; -0.87, p <0.001), than those who did not report discrimination

Prospective associations between perceived disability discrimination and wellbeing

In prospective analyses (second panel Table 2), those who reported perceived disability discrimination had higher levels of psychological distress 4 years later than those who did not report discrimination, independent of covariates and baseline psychological distress (B = 2.88, 95% CI 1.39; 4.36, p <0.001). We detected a prospective association between perceived disability discrimination and poorer SF-12 mental functioning (B = -5.12; 95% CI -8.91; -1.34, p =0.008). Those who reported disability discrimination had slightly lower life satisfaction (means= 4.14 vs 4.67) and a greater proportion rated their health as fair/poor (67.3% vs 62.1%) than those who did not report discrimination at follow-up. However, these differences did not reach statistical significance.

Sensitivity analyses

For peer review only - http://bmjopen.bmj.com/site/about/guidelines.xhtml

In the first sensitivity analysis (Table 3), cross-sectional findings for those who provided complete data at wave 5 were similar to the full-sample at wave 1. The demographic characteristics of those lost to follow-up were similar to those of complete cases (Table 4). Only education differed significantly between the groups, with those who provided complete data at wave 5 more likely to hold a degree (27.0%) than those lost to follow-up (19.7%).

In the second sensitivity analysis, removing each of the discriminatory behaviours from the measure of discrimination in turn did not alter the cross-sectional results (Table 5). Prospectively, the association between perceived disability discrimination and increased psychological distress remained the same regardless of the type of discriminatory behaviour removed from the measure. For SF-12 mental functioning, the association was fairly robust to the type of discriminatory behaviour, but was slightly attenuated when "feeling unsafe" was removed from the discrimination variable (p = 0.058). Again, no significant prospective associations were detected for life satisfaction and self-rated health.

Discussion

In a sample of UK-based participants with self-reported disability, perceived discrimination was associated with higher prevalence of depression, greater psychological distress, and poorer mental functioning, life satisfaction and self-rated health. Prospectively, disability discrimination was associated with increased psychological distress and worse mental functioning four-years later. Our results were robust to adjustment for a range of covariates and were not driven by any specific kind of discriminatory behaviour. No significant prospective relationships with life satisfaction and self-rated health were observed.

Previous literature has been dominated by cross-sectional studies. To our knowledge, only one previous study has investigated the prospective association between disability discrimination and wellbeing outcomes. In this analysis of US adults from the HRS cohort,

For peer review only - http://bmjopen.bmj.com/site/about/guidelines.xhtml

perceived disability discrimination was associated with poorer life satisfaction and self-rated health over four year follow-up [26]. In the current study we observed poorer mental functioning and greater psychological distress four years later in those who reported disability discrimination, taking into account baseline scores on these variables. We failed to detect a significant association between perceived discrimination and life satisfaction or self-rated health at follow-up. Although on average, those who perceived disability discrimination in our sample had poorer life satisfaction and were more likely to rate their health as fair/poor at follow-up than those who did not perceive discrimination, these differences did not reach statistical significance. One reason for the divergence in findings between our study and the HRS analysis [26] may be study design. We limited our analyses to those with a confirmed disability, whereas in the HRS study associations between wellbeing and disability discrimination were assessed across the entire sample. Our analysis offers more precision in the assessment of the relationship between disability discrimination and wellbeing outcomes, by directly comparing people with disability who did and did not perceive discrimination. Another possibility for these null findings may be that significant associations between discrimination and life satisfaction and self-rated health do not become apparent until older adulthood, perhaps allowing for repeated exposures to disability discrimination. However, this assertion remains to be tested. Another potential explanation is that the impact of ongoing disability discrimination on life satisfaction and self-rated health in our sample had already become apparent at the time of the baseline survey, limiting the scope for further decline.

Our study adds to the cross-sectional literature linking perceived disability discrimination and poorer mental health and wellbeing outcomes by demonstrating associations in a community sample of disabled people living in the UK. Our results extend the findings of an earlier study linking stigma and depression in those with intellectual disability[17], by establishing this relationship in a sample with a broader range of disability.

For peer review only - http://bmjopen.bmj.com/site/about/guidelines.xhtml

In keeping with previous studies, we observed greater psychological distress[18–20] and poorer life satisfaction[21–23,26] in those who reported disability discrimination. Our study adds to this existing evidence by demonstrating this link in a UK-based sample for the first time. Similar to earlier work from Australian, European and North American samples[19,24,26,36], we observed a relationship between perceived disability discrimination and poorer self-rated health. Cross-sectional studies cannot determine whether perceived disability discrimination predicts poor mental wellbeing, or whether perceptions of discrimination are an indicator of psychological distress. Our prospective findings therefore add to the field in establishing that perceived disability discrimination predicts psychological distress and poorer mental functioning, net of baseline associations, so has negative implications for future mental health.

This is an observational study and longitudinal analyses do not necessarily imply causality. There could be unmeasured factors responsible for the associations that emerged. Nevertheless, with regard to the pathways linking perceived disability discrimination and mental health and wellbeing, there are several possibilities that could explain our results. One mechanism could be that perceptions of disability discrimination in healthcare settings serve to impede access to health services. An analysis of HRS found that reports of frequent discrimination in healthcare settings were predictive of new or worsened disability over four year follow-up[37]. Quantitative[38,39] and qualitative[40] evidence suggests that those who perceive disability discrimination are less likely to seek healthcare. However, there may be sex differences in this association, with a Swedish study only detecting a relationship between disability discrimination and healthcare avoidance in women[39].However, no interaction between sex and perceived discrimination was detected in the current study (data not shown).

Poor health behaviours are another potential mechanism linking disability discrimination and poorer mental wellbeing. For example, perceived disability discrimination

For peer review only - http://bmjopen.bmj.com/site/about/guidelines.xhtml

has been linked with worse sleep quality in the HRS, with psychological distress acting as a full mediator of this association[41]. It is possible that disabled people could engage in negative health behaviours as a means of coping with the psychological impact of discrimination. In a study of 304 individuals with disability, perceived disability discrimination was positively associated with illicit drug use[42]. Eating may offer a source of comfort in the face of discrimination[43]. A US study of over 5000 individuals, observed a link between physical disability discrimination and overeating[44].

Another possibility is that perceived disability discrimination and mental wellbeing are linked through disturbed stress-related biological processes. In line with the theory of allostatic load, perceived chronic discrimination causing frequent activation of the stress response system, could over time result in disturbances across multiple biological systems[45]. Systematic reviews and meta-analyses, which have predominately focused on racism, suggest that discrimination is linked with heightened cardiovascular stress reactivity[14,46], while race[46–48] and weight discrimination[49] have been linked with alterations in cortisol. To our knowledge, no study has investigated associations between perceived disability discrimination and changes in cardiovascular or neuroendocrine activity. In the HRS cohort, perceived disability discrimination was linked with raised c-reactive protein levels cross-sectionally[50]. Heightened inflammation is thought to be predictive of poorer mental wellbeing[51], offering a plausible pathway between perceived disability discrimination and later psychological distress and poorer mental functioning seen in the present study. Further work is required to confirm this assertion, particularly as the HRS analysis was not limited to those with a confirmed disability.

Our study had several strengths. The use of the UKHLS cohort allowed us to examine changes in mental wellbeing over a 4-year period across a wide age range (17 – 96 years), while adjusting statistically for factors that could confound associations. The discrimination

For peer review only - http://bmjopen.bmj.com/site/about/guidelines.xhtml

measure took into account several kinds of discriminatory behaviour and included multiple settings where perceived disability discrimination could be encountered.

However, the study was not without limitations. Our findings are based on perceptions of disability discrimination rather than objective encounters with disability discrimination. It is possible that perceiving oneself as a target for discrimination and objective encounters with discrimination could have differing consequences for mental wellbeing. Indeed, earlier work in a sample with significant health limitations, indicates that individuals with poorer mental wellbeing may be more likely to perceive stigma[52]. Future studies assessing reciprocal prospective associations between perceived disability discrimination and health could help to clarify this issue. Our discrimination measure was based on self-reports of experiences during the past 12 months and was therefore subject to recall bias. Further, this measure was not specific to disability discrimination. The fact that participants were able to attribute multiple reasons for their experience of discrimination, could have helped avoid priming or bias. Other tools specifically designed to assess disability discrimination could have garnered different results. Further work is required to understand how disability discrimination interacts with other types of discrimination to influence wellbeing. Disability discrimination was only assessed at one point in time, meaning our measure does not necessarily reflect pervasive discrimination. Future research is required to determine whether perceptions of disability discrimination are persistent or alter over time. Our study included participants with physical, cognitive and sensory disabilities. However, for a large proportion (37%) their disability type was unknown and classified as “other”, limiting our understanding.

Overall, our study adds to the literature by demonstrating prospective associations between perceived disability discrimination and mental wellbeing outcomes. These findings emphasise the need to reduce the prevalence of disability discrimination, with the benefit of promoting equality as well as possible advantages for mental wellbeing too. Though complete

For peer review only - http://bmjopen.bmj.com/site/about/guidelines.xhtml

elimination of disability discrimination is likely to be difficult, recognition of disability discrimination as an issue is the first step in preventing its occurrence. Addressing this could involve raising awareness through the use of campaigns. The Public Sector Equality Duty in the UK requires public bodies to have due regard to the need to eliminate discrimination and this awareness raising should begin early in life[7]. However, it is estimated that less than 40% of English primary schools have a disability equality scheme in place, with race and gender equality more often prioritised over disability equality[53]. Therefore, further effort on this issue is required[7], particularly as disability discrimination is perceived to be more widespread than gender discrimination in Europe[8].

As well as macro-level awareness raising, on an individual basis the negative impact of perceived disability discrimination on mental health may be buffered through the use of social support. In two cross-sectional studies of US adults with varied disability diagnoses, those with more friends reported greater life satisfaction and these friendships attenuated the link between functional impairment and poorer quality of life[54]. In an Israeli study, perceived disability discrimination and poorer life satisfaction were only linked in those with low and moderate levels of social support, with no association in those with greater levels of support[55]. Further research into the mechanisms underlying disability discrimination is necessary to develop awareness campaigns and to appropriately target individual-level interventions.

For peer review only - http://bmjopen.bmj.com/site/about/guidelines.xhtml

Author contributions: RAH conducted the statistical analysis and wrote the manuscript. AS edited and reviewed the manuscript. RPL edited and reviewed the manuscript. SJ provided scientific overview, edited and reviewed the manuscript.

Patient and Public Involvement

No patients or members of the public were involved in study design or conduct.

For peer review only

For peer review only - http://bmjopen.bmj.com/site/about/guidelines.xhtml

References

1 The Equality Act. 2010. https://www.legislation.gov.uk/ukpga/2010/15/contents

2 World Health Organisation. *World report on disability 2011*. World Health Organisation 2011.

3 Leonardi M, Bickenbach J, Ustun TB, *et al.* The definition of disability: what is in a name? *The Lancet* 2006;**368**:1219–21. doi:10.1016/S0140-6736(06)69498-1

4 Department for Work and Pensions. Family Resources Survey 2017-18. 2019.https://assets.publishing.service.gov.uk/government/uploads/system/uploads/attachment_data/file/790000/family-resources-survey-2017-18.pdf

5 Equality and Human Rights Commission. Being disabled in Britain: A journey less equal. 2017.

6 Office for National Statistics. Life Opportunities Survey: Wave 3 results. 2015.https://www.gov.uk/government/statistics/life-opportunities-survey-wave-3-results

7 Equality and Human Rights Commission. Hidden in plain sight: inquiry into disability-related harassment. 2011.https://www.equalityhumanrights.com/en/publication-download/hidden-plain-sight-inquiry-disability-related-harassment (accessed 28 Mar 2019).

8 European Union. *Discrimination in the EU in 2015*. 2015.

9 Sykes W, Groom C, Desai P. Disability-related harassment: the role of public bodies A qualitative research report. Manchester: : Equality and Human Rights Commission 2011.

10 Office for National Statistics. Crime Survey for England and Wales 2014-2015. 2016.

11 Scottish Government. Report of Independent Advisory Group on Hate Crime, Prejudice and Community Cohesion'. 2016.https://www.gov.scot/publications/report-independent-advisory-group-hate-crime-prejudice-community-cohesion/

12 Corcoran H, Lader D, Smith K. Hate crime, England and Wales, 2014 to 2015. 2015.

13 Coleman N, Sykes W, Groom C. Barriers to employment and unfair treatment at work: a quantitative analysis of disabled people's experiences. Equality and Human Rights Commission 2013.

14 Pascoe EA, Smart Richman L. Perceived discrimination and health: a meta-analytic review. *Psychol Bull* 2009;**135**:531–54. doi:10.1037/a0016059

15 Goto JB, Couto PFM, Bastos JL. [Systematic review of epidemiological studies on interpersonal discrimination and mental health]. *Cad Saude Publica* 2013;**29**:445–59.

For peer review only

16 Schmitt MT, Branscombe NR, Postmes T, *et al.* The consequences of perceived discrimination for psychological well-being: a meta-analytic review. -. *Psychol Bull* 2014;**140**:921–48.

17 Ali A, King M, Strydom A, *et al.* Self-reported stigma and symptoms of anxiety and depression in people with intellectual disabilities: Findings from a cross sectional study in England. *J Affect Disord* 2015;**187**:224–31. doi:10.1016/j.jad.2015.07.046

18 Temple JB, Kelaher M. Is disability exclusion associated with psychological distress? Australian evidence from a national cross-sectional survey. *BMJ Open* 2018;**8**:e020829. doi:10.1136/bmjopen-2017-020829

19 Krnjacki L, Priest N, Aitken Z, *et al.* Disability-based discrimination and health: findings from an Australian-based population study. *Aust N Z J Public Health* 2018;**42**:172–4.

20 Wamala S, Bostrom G, Nyqvist K. Perceived discrimination and psychological distress in Sweden. *Br J Psychiatry* 2007;**190**:75–6.

21 Daley A, Phipps S, Branscombe NR. The social complexities of disability: Discrimination, belonging and life satisfaction among Canadian youth. *SSM - Popul Health* 2018;**5**:55–63. doi:10.1016/j.ssmph.2018.05.003

22 Kim S. A Study of the Impact of Discrimination Experience on Life Satisfaction in Korean Women with Severe Disabilities. *World Acad Sci Eng Technol Int J Soc Behav Educ Econ Bus Ind Eng* 2015;**9**:3205–9.

23 Kagan M, Itzick M, Tal-Katz P. Demographic, Psychosocial, and Health- and Disability-Related Factors Associated With Psychological Distress Among People With Physical Disabilities. *Rehabil Psychol* 2018;**63**:392–9. doi:10.1037/rep0000206

24 Du Mont J, Forte T. Perceived discrimination and self-rated health in Canada: an exploratory study. *BMC Public Health* 2016;**16**:742. doi:10.1186/s12889-016-3344-y

25 Alvarez-Galvez J, Salvador-Carulla L. Perceived discrimination and self-rated health in Europe: evidence from the European Social Survey (2010). *PloS One* 2013;**8**:e74252. doi:10.1371/journal.pone.0074252

26 Sutin AR, Stephan Y, Carretta H, *et al.* Perceived discrimination and physical, cognitive, and emotional health in older adulthood. *Am J Geriatr Psychiatry Off J Am Assoc Geriatr Psychiatry* 2015;**23**:171–9. doi:10.1016/j.jagp.2014.03.007

27 University of Essex. Institute for Social and Economic Research, NatCen Social Research, Kantar Public. *Understanding Society: Waves 1-7, 2009-2016 and Harmonised BHPS: Waves 1-18, 1991-2009.* 9th Edition. UK data service 2017.

28 McFall S, Nandi A, Platt L. *Understanding Society: UK Household Longitudinal Study: User Guide to Ethnicity and Immigration Research.* 4th ed. Colchester: : University of Essex 2017.

29 Knies G. *Understanding society: The UK household longitudinal study, Waves 1–7 (User Guide).* University of Essex: : Colchester: Institute for Social and Economic Research 2017.

For peer review only - http://bmjopen.bmj.com/site/about/guidelines.xhtml

30 Goldberg D, Williams P. *A user's guide to the General Health Questionnaire.* Basingstoke: : NFER-Nelson 1988.

31 Ware J, Kosinski M, Keller SD. A 12-Item Short-Form Health Survey: construction of scales and preliminary tests of reliability and validity. *Med Care* 1996;**34**:220–33.

32 Ware JE. *How to score version 2 of the SF-12 health survey (with a supplement documenting version 1) Edition: first.* Lincoln, RI: : Health Assessment Lab 2002.

33 Steptoe A, Jackson SE. The Life Skills of Older Americans: Association with Economic, Psychological, Social, and Health Outcomes. *Sci Rep* 2018;**8**:9669. doi:10.1038/s41598-018-27909-w

34 DeSalvo KB, Bloser N, Reynolds K, *et al.* Mortality prediction with a single general self-rated health question. A meta-analysis. *J Gen Intern Med* 2006;**21**:267–75. doi:10.1111/j.1525-1497.2005.00291.x

35 OECD. *What are equivalence scales?* 2005.

36 Alvarez A, Faccioli J, Guinzbourg M, *et al.* Endocrine and inflammatory profiles in type 2 diabetic patients with and without major depressive disorder. *BMC Res Notes* 2013;**6**:61. doi:10.1186/1756-0500-6-61

37 Rogers SE, Thrasher AD, Miao Y, *et al.* Discrimination in Healthcare Settings is Associated with Disability in Older Adults: Health and Retirement Study, 2008-2012. *J Gen Intern Med* 2015;**30**:1413–20. doi:10.1007/s11606-015-3233-6

38 Moscoso-Porras MG, Alvarado GF. Association between perceived discrimination and healthcare-seeking behavior in people with a disability. *Disabil Health J* 2018;**11**:93–8. doi:10.1016/j.dhjo.2017.04.002

39 Wamala S, Merlo J, Boström G, *et al.* Perceived discrimination, socioeconomic disadvantage and refraining from seeking medical treatment in Sweden. *J Epidemiol Community Health* 2007;**61**:409–15. doi:10.1136/jech.2006.049999

40 Ali A, Scior K, Ratti V, *et al.* Discrimination and other barriers to accessing health care: perspectives of patients with mild and moderate intellectual disability and their carers. *PloS One* 2013;**8**:e70855. doi:10.1371/journal.pone.0070855

41 Vaghela P, Sutin AR. Discrimination and sleep quality among older US adults: the mediating role of psychological distress. *Sleep Health J Natl Sleep Found* 2016;**2**:100–8. doi:10.1016/j.sleh.2016.02.003

42 Li Li DM. disability and illicit drug use: an application of labeling theory. *Deviant Behav* 2001;**22**:1–21. doi:10.1080/016396201750065784

43 Wansink B, Cheney MM, Chan N. Exploring comfort food preferences across age and gender. *Physiol Behav* 2003;**79**:739–47.

44 Sutin A, Robinson E, Daly M, *et al.* Weight discrimination and unhealthy eating-related behaviors. *Appetite* 2016;**102**:83–9. doi:10.1016/j.appet.2016.02.016

For peer review only - http://bmjopen.bmj.com/site/about/guidelines.xhtml

45 McEwen BS. Stress, adaptation, and disease. Allostasis and allostatic load. *Ann N Y Acad Sci* 1998;**840**:33–44.

46 Lockwood KG, Marsland AL, Matthews KA, *et al.* Perceived discrimination and cardiovascular health disparities: a multisystem review and health neuroscience perspective. *Ann N Y Acad Sci* 2018;**1428**:170–207. doi:10.1111/nyas.13939

47 Busse D, Yim IS, Campos B, *et al.* Discrimination and the HPA axis: current evidence and future directions. *J Behav Med* 2017;**40**:539–52. doi:10.1007/s10865-017-9830-6

48 Korous KM, Causadias JM, Casper DM. Racial discrimination and cortisol output: A meta-analysis. *Soc Sci Med 1982* 2017;**193**:90–100. doi:10.1016/j.socscimed.2017.09.042

49 Jackson SE, Kirschbaum C, Steptoe A. Perceived weight discrimination and chronic biochemical stress: A population-based study using cortisol in scalp hair. *Obes Silver Spring Md* 2016;**24**:2515–21. doi:10.1002/oby.21657

50 Sutin AR, Stephan Y, Luchetti M, *et al.* Perceived weight discrimination and C-reactive protein. *Obes Silver Spring Md* 2014;**22**:1959–61. doi:10.1002/oby.20789

51 Slavich GM, Irwin MR. From stress to inflammation and major depressive disorder: a social signal transduction theory of depression. *Psychol Bull* 2014;**140**:774–815. doi:10.1037/a0035302

52 Alonso J, Buron A, Bruffaerts R, *et al.* Association of perceived stigma and mood and anxiety disorders: results from the World Mental Health Surveys. *Acta Psychiatr Scand* 2008;**118**:305–14.

53 Beckett AE, Buckner L. Promoting positive attitudes towards disabled people: definition of, rationale and prospects for anti-disablist education. *Br J Sociol Educ* 2012;**33**:873–91. doi:10.1080/01425692.2012.692046

54 Silverman AM, Molton IR, Smith AE, *et al.* Solace in solidarity: Disability friendship networks buffer well-being. *Rehabil Psychol*;**62**:525–33.

55 Itzick M, Kagan M, Tal-Katz P. Perceived social support as a moderator between perceived discrimination and subjective well-being among people with physical disabilities in Israel. *Disabil Rehabil* 2018;**40**:2208–16. doi:10.1080/09638288.2017.1331380

For peer review only - http://bmjopen.bmj.com/site/about/guidelines.xhtml

Table 1 Associations between perceived disability discrimination and sociodemographic factors at wave 1 (2009/10)

	**No perceived discrimination** (***n*** **= 754)**	**Perceived discrimination** (***n*** **= 117)**	***p***
Age (years)	53.42 (16.56)	48.29 (14.89)	0.001
17-34	100 (13.3%)	20 (17.1%)	
35-44	149 (19.8%)	23 (19.7%)	
45-54	150 (19.9%)	34 (29.1%)	
55-64	134 (17.8%)	23 (19.7%)	
65+	221 (29.3%)	17 (14.5%)	
Sex (% men)	334 (44.3%)	54 (46.2%)	0.707
Household income (£)	1123.28 (930.47)	1087.07 (698.65)	0.687
£0-499	105 (13.9%)	12 (10.3%)	
£500-999	314 (41.6%)	55 (47.0%)	
£1000-1499	189 (25.1%)	27 (23.1%)	
£1500-1999	77 (10.2%)	13 (11.1%)	
£2000+	69 (9.2%)	10 (8.5%)	
Education (% yes)			0.003
University Degree	171 (22.7%)	33 (28.2%)	-
School qualification	285 (37.8%)	57 (48.7%)	-
No qualification	298 (39.5%)	27 (23.1%)	-
Ethnicity			0.002
White	153 (20.3%)	32 (27.4%)	-
South Asian	333 (44.2%)	36 (30.8%)	-
Black	168 (22.3%)	21 (17.9%)	-

For peer review only - http://bmjopen.bmj.com/site/about/guidelines.xhtml

Other	100 (13.3%)	28 (23.9%)	-
Disability type			< 0.001
Physical	351 (46.6%)	16 (13.7%)	
Sensory	84 (11.1%)	11 (9.4%)	
Cognitive	73 (9.7%)	14 (12.0%)	
Other	246 (32.6%)	76 (65.0%)	

Data are presented as means (SD) and n (%)

For peer review only

Table 2: Cross-sectional and prospective associations between perceived disability discrimination and emotional wellbeing outcomes

		Cross-sectional				Prospective			
		n	No perceived discrimination	n	Perceived discrimination	n	No perceived discrimination	n	Perceived discrimination
Depression									
	% (SE)	751	9.3 (0.11)	117	38.4 (0.03)	-	-	-	-
	OR [95%CI]		Ref		5.40 [3.25; 8.97]***		-	-	-
Psychological distress									
	Mean score (SE)	454	2.93 (0.17)	82	6.21 (0.40)	177	2.53 (0.26)	31	5.40 (0.68)
	Coeff. [95%CI]		Ref		3.28 [2.41; 4.14]***		Ref		2.88 [1.39; 4.36]***
SF-12 mental									
	Mean score (SE)	742	44.87 (0.42)	117	37.57 (1.10)	239	45.24 (0.69)	43	40.12 (1.74)
	Coeff. [95%CI]		Ref		-7.35 [-9.70; -5.02]***		Ref		-5.12 [-8.91; -1.34]**
Life satisfaction									
	Mean score (SE)	454	4.69 (0.08)	84	3.42 (0.18)	171	4.67 (0.12)	34	4.14 (0.29)
	Coeff. [95%CI]		Ref		-1.27 [-1.66; -0.87]***		Ref		-0.53 [-1.18; 0.11]
Fair/poor self-rated health									
	% (SE)	754	69.7 (0.01)	117	80.7 (0.04)	385	62.1 (0.02)	55	67.3 (0.06)
	OR [95%CI]		1.00 (Ref)		2.05 [1.19; 3.51]**		1.00 (Ref)		1.29 [0.59; 2.83]

All analyses are adjusted for age, sex, household income, education, ethnicity and disability type. Prospective analyses are additionally adjusted for baseline wellbeing status/score.
Coeff = unstandardized B coefficient (white rows), CI = confidence interval, OR = odds ratio (grey rows), SE = standard error.
*p<0.05, **p<0.01, ***p <0.001

Possible scores on the psychological distress scale range from 0-12, SF-12 mental component scale range from 0-100 and the life satisfaction scale scores range from 0-7.

For peer review only - http://bmjopen.bmj.com/site/about/guidelines.xhtml

Table 3: Cross-sectional and prospective associations between perceived disability discrimination and emotional wellbeing outcomes (complete cases at wave 5)

		Cross-sectional				Prospective			
		n	No perceived discrimination	n	Perceived discrimination	n	No perceived discrimination	n	Perceived discrimination
Psychological distress									
	Mean score (SE)	177	2.81 (0.26)	31	5.46 (0.66)	177	2.53 (0.26)	31	5.40 (0.68)
	Coeff. [95%CI]		Ref		2.65 [1.21; 4.08]***		Ref		2.88 [1.39; 4.36]***
SF-12 mental									
	Mean score (SE)	239	45.89 (0.71)	43	38.69 (1.76)	239	45.24 (0.69)	43	40.12 (1.74)
	Coeff. [95%CI]		Ref		-7.20 [-11.01; -3.39]***		Ref		-5.12 [-8.91; -1.34]**
Life satisfaction									
	Mean score (SE)	171	4.78 (0.12)	34	3.50 (0.29)	171	4.67 (0.12)	34	4.14 (0.29)
	Coeff. [95%CI]		Ref		-1.27 [-1.91; -0.63]***		Ref		-0.53 [-1.18; 0.11]
Fair/poor self-rated health									
	% (SE)	385	66.5 (0.02)	55	81.6 (0.06)	385	62.1 (0.02)	55	67.3 (0.06)
	OR [95%CI]		1.00 (Ref)		2.66 [1.16; 6.08]*		1.00 (Ref)		1.29 [0.59; 2.83]

All analyses are adjusted for age, sex, household income, education, ethnicity and disability type. Prospective analyses are additionally adjusted for baseline wellbeing status/score.
Coeff = unstandardized B coefficient (white rows), CI = confidence interval, OR = odds ratio (grey rows), SE = standard error.
*p<0.05, **p<0.01, ***p <0.001

Possible scores on the psychological distress scale range from 0-12, SF-12 mental component scale range from 0-100 and the life satisfaction scale scores range from 0-7.

For peer review only - http://bmjopen.bmj.com/site/about/guidelines.xhtml

For peer review only

Table 4 Participant characteristics at wave 1 (2009/10) of complete cases and those lost to follow-up

	Lost to follow-up (n=431)	Complete cases (n=440)	*p*
Age (years)	52.26 (17.08)	53.19 (15.76)	0.407
17-34	25 (5.8%)	12 (2.7%)	
35-44	46 (10.7%)	37 (8.4%)	
45-54	79 (18.4%)	93 (21.1%)	
55-64	91 (21.2%)	93 (21.1%)	
65+	189 (44.0%)	205 (46.6%)	
Sex (% men)	200 (46.4%)	188 (42.7%)	0.275
Household income (£)	1101.26 (1037.11)	1135.22 (748.46)	0.579
£0-499	69 (16.0%)	48 (10.9%)	
£500-999	175 (40.6%)	194 (44.1%)	
£1000-1499	113 (26.2%)	103 (23.4%)	
£1500-1999	42 (9.7%)	48 (10.9%)	
£2000+	32 (7.4%)	47 (10.7%)	
Education (% yes)			0.024
University Degree	85 (19.7%)	119 (27.0%)	-
School qualification	171 (39.7%)	171 (38.9%)	-
No qualification	175 (40.6%)	150 (34.1%)	-
Ethnicity			0.213
White	88 (20.4%)	97 (22.0%)	-
South Asian	172 (39.9%)	197 (44.8%)	-

For peer review only - http://bmjopen.bmj.com/site/about/guidelines.xhtml

Black	105 (24.4%)	84 (19.1%)	-
Other	66 (15.3%)	62 (14.1%)	-
Disability type			0.189
Physical	166 (38.5%)	201 (45.7%)	
Sensory	51 (11.8%)	44 (10.0%)	
Cognitive	47 (10.9%)	40 (9.1%)	
Other	167 (38.7%)	155 (35.2%)	
Mental health/wellbeing			
Psychological distress	3.46 (3.85)	3.41 (3.86)	0.874
SF-12	43.72 (12.93)	44.01 (12.30)	0.741
Life satisfaction	4.40 (1.75)	4.56 (1.69)	0.306
Self-rated health (% fair/poor)	319 (74.0%)	301 (68.4%)	0.068

Data are presented as means (SD) and n (%)

*Complete cases are defined as those who were present at wave 1 and provided data on at least one wellbeing measure at wave 5.

For peer review only

Table 5: Sensitivity analysis: Perceived disability discrimination measure excluding each discriminatory behaviour in turn

**Cross-sectional analyses (wave 1)**		**Model 1**	**Model 2**	**Model 3**	**Model 4**
Depression	OR [95%CI]	4.41 (2.55;7.60)***	5.24 (3.06;8.98)***	4.80 (2.92;7.88)***	5.41 (3.26;8.98)***
Psychological distress	Coeff. [95%CI]	3.64 (2.68;4.60)***	3.28 (2.33;4.23)***	3.13 (2.26;4.01)***	3.27 (2.41;4.14)***
SF-12 mental	Coeff. [95%CI]	-6.63 (-9.29;-3.97)***	-7.61 (-10.15;-5.08)***	-7.53 (-9.89;-5.18)***	-7.35 (-9.68;-5.02)***
Life satisfaction	Coeff. [95%CI]	-1.23 (-1.69;-0.76)***	-1.45 (-1.88;-1.02)***	-1.24 (-1.64;-0.85)***	-1.27 (-1.66;-0.87)***
Fair/poor self-rated health	OR [95%CI]	1.92 (1.04;3.53)*	2.39 (1.32;4.33)**	2.21 (1.28;3.81)**	2.04 (1.19;3.50)**
**Prospective analyses (wave 5)**		**Model 1**	**Model 2**	**Model 3**	**Model 4**
Psychological distress	Coeff. [95%CI]	2.78 (1.14;4.41)***	2.69 (1.15;4.24)***	2.89 (1.45;4.33)***	2.88 (1.39;4.36)***
SF-12 mental	Coeff. [95%CI]	-4.33 (-8.81;0.14)	-4.90 (-8.86;-0.94)**	-5.94 (-9.59;-2.28)**	-5.13 (-8.91;-1.34)**
Life satisfaction	Coeff. [95%CI]	-0.39 (-1.14;0.36)	-0.37 (-1.06;0.32)	-0.39 (-1.02;0.25)	-0.53 (-1.18;0.11)
Fair/poor self-rated health	OR [95%CI]	1.23 (0.50;3.03)	1.30 (0.57;2.95)	1.31 (0.61;2.83)	1.29 (0.59;2.83)

All analyses are adjusted for age, sex, household income, education, ethnicity and disability type. Prospective analyses are additionally adjusted for baseline wellbeing status/score.

Model 1 excludes "*felt unsafe at some place*" from the measure of perceived gender discrimination; Model 2 excludes "*avoided some place* "; Model 3 excludes ""*was insulted at some place* "; and Model 4 excludes "*was attacked at some place*"

Coeff = unstandardized B coefficient (white rows), CI = confidence interval, OR = odds ratio (grey rows), SE = standard error.

**p*<0.05, ***p*<0.01, ****p*<0.001

Possible scores on the psychological distress scale range from 0-12, SF-12 mental component scale range from 0-100 and the life satisfaction scale scores range from 0-7.

For peer review only

Supplementary table 1: Perceived disability discrimination types and settings by type of disability

**Types of discrimination**	**Physical**	**Sensory**	**Cognitive**	**Other**	**p value**
Felt unsafe at someplace	12 (85.7%)	9 (90%)	12 (92.3%)	60 (84.5%)	0.874
Avoided at someplace	8 (53.3%)	6 (75%)	9 (75%)	52 (69.3%)	0.336
Felt insulted at someplace	3 (25%)	1 (12.5%)	1 (10%)	15 (27.3%)	0.572
Attacked at someplace	0 (0%)	0 (0%)	0(0%)	3(4.4%)	0.621
**Settings**					
School/work	2 (12.5%)	1 (9.1%)	0 (0%)	12 (15.8%)	0.424
Public transport	10 (62.5%)	3 (27.3%)	6 (42.9%)	41 (53.9%)	0.266
Bus or train stations	5 (31.3%)	2 (18.2%)	6 (42.9%)	34 (44.7%)	0.328
Taxis	4 (25%)	0 (0%)	1 (7.1%)	9 (11.8%)	0.223
Public buildings	8 (50%)	7 (63.6%)	7 (50%)	48 (63.2%)	0.654
On the street	12 (75%)	8 (72.7%)	9 (64.3%)	62 (81.6%)	0.504
At home	3 (18.8%)	3 (27.3%)	4 (28.6%)	20 (26.3%)	0.920

Data are presented as numbers (% yes)

For peer review only

Reporting checklist for cohort study.

Based on the STROBE cohort guidelines.

Instructions to authors

Complete this checklist by entering the page numbers from your manuscript where readers will find each of the items listed below.

Your article may not currently address all the items on the checklist. Please modify your text to include the missing information. If you are certain that an item does not apply, please write "n/a" and provide a short explanation.

Upload your completed checklist as an extra file when you submit to a journal.

In your methods section, say that you used the STROBE cohortreporting guidelines, and cite them as:

von Elm E, Altman DG, Egger M, Pocock SJ, Gotzsche PC, Vandenbroucke JP. The Strengthening the Reporting of Observational Studies in Epidemiology (STROBE) Statement: guidelines for reporting observational studies.

For peer review only

		Reporting Item	Page Number
**Title and abstract**			
Title	#1a	Indicate the study's design with a commonly used term in the title or the abstract	1
Abstract	#1b	Provide in the abstract an informative and balanced summary	2

		of what was done and what was found	
**Introduction**			
Background / rationale	#2	Explain the scientific background and rationale for the investigation being reported	4
Objectives	#3	State specific objectives, including any prespecified hypotheses	6
**Methods**			
Study design	#4	Present key elements of study design early in the paper	6
6Setting	#5	Describe the setting, locations, and relevant dates, including periods of recruitment, exposure, follow-up, and data collection	6
Eligibility criteria	#6a	Give the eligibility criteria, and the sources and methods of selection of participants. Describe methods of follow-up.	6
Eligibility criteria	#6b	For matched studies, give matching criteria and number of exposed and unexposed	N/A
Variables	#7	Clearly define all outcomes, exposures, predictors, potential confounders, and effect modifiers. Give diagnostic criteria, if applicable	7-8
Data sources / measurement	#8	For each variable of interest give sources of data and details of methods of assessment (measurement). Describe comparability of assessment methods if there is more than one group. Give information separately for for exposed and unexposed groups if applicable.	7-8

For peer review only

Bias	#9	Describe any efforts to address potential sources of bias	8
Study size	#10	Explain how the study size was arrived at	6
Quantitative variables	#11	Explain how quantitative variables were handled in the analyses. If applicable, describe which groupings were chosen, and why	9
Statistical methods	#12a	Describe all statistical methods, including those used to control for confounding	9
Statistical methods	#12b	Describe any methods used to examine subgroups and interactions	9
Statistical methods	#12c	Explain how missing data were addressed	6
Statistical methods	#12d	If applicable, explain how loss to follow-up was addressed	6
Statistical methods	#12e	Describe any sensitivity analyses	9-10
**Results**			
Participants	#13a	Report numbers of individuals at each stage of study—eg numbers potentially eligible, examined for eligibility, confirmed eligible, included in the study, completing follow-up, and analysed. Give information separately for for exposed and unexposed groups if applicable.	6
Participants	#13b	Give reasons for non-participation at each stage	N/A

For peer review only

Participants	#13c	Consider use of a flow diagram	N/A
Descriptive data	#14a	Give characteristics of study participants (eg demographic, clinical, social) and information on exposures and potential confounders. Give information separately for exposed and unexposed groups if applicable.	10
Descriptive data	#14b	Indicate number of participants with missing data for each variable of interest	6
Descriptive data	#14c	Summarise follow-up time (eg, average and total amount)	6
Outcome data	#15	Report numbers of outcome events or summary measures over time. Give information separately for exposed and unexposed groups if applicable.	N/A
Main results	#16a	Give unadjusted estimates and, if applicable, confounder-adjusted estimates and their precision (eg, 95% confidence interval). Make clear which confounders were adjusted for and why they were included	11
Main results	#16b	Report category boundaries when continuous variables were categorized	9
Main results	#16c	If relevant, consider translating estimates of relative risk into absolute risk for a meaningful time period	N/A
Other analyses	#17	Report other analyses done—e.g., analyses of subgroups and interactions, and sensitivity analyses	12

Discussion

For peer review only

Key results	#18	Summarise key results with reference to study objectives	12
Limitations	#19	Discuss limitations of the study, taking into account sources of potential bias or imprecision. Discuss both direction and magnitude of any potential bias.	16
Interpretation	#20	Give a cautious overall interpretation considering objectives, limitations, multiplicity of analyses, results from similar studies, and other relevant evidence.	12-14
Generalisability	#21	Discuss the generalisability (external validity) of the study results	16-17

Other Information

Funding	#22	Give the source of funding and the role of the funders for the present study and, if applicable, for the original study on which the present article is based	1

None The STROBE checklist is distributed under the terms of the Creative Commons Attribution License CC-BY. This checklist can be completed online using https://www.goodreports.org/, a tool made by the EQUATOR Network in collaboration with Penelope.ai

For peer review only

BMJ Open

Disability discrimination and wellbeing: A prospective analysis

Journal:	*BMJ Open*
Manuscript ID	bmjopen-2019-035714.R1
Article Type:	Original research
Date Submitted by the Author:	20-Jan-2020
Complete List of Authors:	Hackett, Ruth; King's College London, Health Psychology Section; University College London, Behavioural Science and Health<br>Steptoe, Andrew; University College London, Behavioural Science and Health<br>Lang, Raymond; University College London, Leonard Cheshire Research Centre, Epidemiology and Public Health<br>Jackson, Sarah ; University College London, Behavioural Science and Health
**Primary Subject Heading**:	Epidemiology
Secondary Subject Heading:	Mental health
Keywords:	EPIDEMIOLOGY, MENTAL HEALTH, PUBLIC HEALTH

SCHOLARONE™
Manuscripts

I, the Submitting Author has the right to grant and does grant on behalf of all authors of the Work (as defined in the below author licence), an exclusive licence and/or a non-exclusive licence for contributions from authors who are: i) UK Crown employees; ii) where BMJ has agreed a CC-BY licence shall apply, and/or iii) in accordance with the terms applicable for US Federal Government officers or employees acting as part of their official duties; on a worldwide, perpetual, irrevocable, royalty-free basis to BMJ Publishing Group Ltd ("BMJ") its licensees and where the relevant Journal is co-owned by BMJ to the co-owners of the Journal, to publish the Work in this journal and any other BMJ products and to exploit all rights, as set out in our licence.

The Submitting Author accepts and understands that any supply made under these terms is made by BMJ to the Submitting Author unless you are acting as an employee on behalf of your employer or a postgraduate student of an affiliated institution which is paying any applicable article publishing charge ("APC") for Open Access articles. Where the Submitting Author wishes to make the Work available on an Open Access basis (and intends to pay the relevant APC), the terms of reuse of such Open Access shall be governed by a Creative Commons licence – details of these licences and which Creative Commons licence will apply to this Work are set out in our licence referred to above.

Other than as permitted in any relevant BMJ Author's Self Archiving Policies, I confirm this Work has not been accepted for publication elsewhere, is not being considered for publication elsewhere and does not duplicate material already published. I confirm all authors consent to publication of this Work and authorise the granting of this licence.

For peer review only

Disability discrimination and wellbeing: A prospective analysis

Ruth A. Hackett[1,2*], Andrew Steptoe[2], Raymond P. Lang[3], and Sarah E. Jackson[2]

[1] Health Psychology Section, Department of Psychology, Institute of Psychiatry, Psychology and Neuroscience, Guy's Hospital, King's College London, London, UK

[2] Department of Behavioural Science and Health, University College London, London, UK

[3] Leonard Cheshire Research Centre, Department of Epidemiology and Public Health, University College London, London, UK

***Corresponding author:** Ruth A. Hackett, PhD. Health Psychology Section, Department of Psychology, Institute of Psychiatry, Psychology and Neuroscience, Guy's Hospital, King's College London, London, UK

Email: ruth.hackett@kcl.ac.uk ORCID: https://orcid.org/0000-0002-5428-2950

Co-author emails: a.steptoe@ucl.ac.uk r.lang@ucl.ac.uk s.e.jackson@ucl.ac.uk

Word count: 4689

Number of tables: 5

For peer review only

For peer review only - http://bmjopen.bmj.com/site/about/guidelines.xhtml

Abstract

Objectives: Disability discrimination is linked with poorer wellbeing cross-sectionally. The aim of this study was to explore prospective associations between disability discrimination and wellbeing.

Design: Prospective cohort study

Setting: The United Kingdom Household Longitudinal Study

Participants: Data were from 871 individuals with a self-reported physical, cognitive or sensory disability.

Primary outcome measures: Depression was assessed in 2009/10. Psychological distress, mental functioning, life satisfaction and self-rated health were assessed in 2009/10 and 2013/14.

Results: Data were analysed using linear and logistic regression with adjustment for age, sex, household income, education, ethnicity and impairment category. Perceived disability discrimination was reported by 117 (13.4%) participants. Cross-sectionally, discrimination was associated with depression (Odds ratio (OR) = 5.40, 95% Confidence Interval (CI) 3.25; 8.97) fair/poor self-rated health (OR=2.05; 95% CI 1.19; 3.51), greater psychological distress (B = 3.28, 95% CI 2.41; 4.14), poorer mental functioning (B = -7.35; 95% CI -9.70; -5.02) and life satisfaction (B = -1.27, 95% CI -1.66; -0.87). Prospectively, discrimination was associated with increased psychological distress (B = 2.88, 95% CI 1.39; 4.36) and poorer mental functioning (B = -5.12; 95% CI -8.91; -1.34), adjusting for baseline scores.

Conclusions: Perceived disability-related discrimination is linked with poorer wellbeing. These findings underscore the need for interventions to combat disability discrimination.

Key words: disability, discrimination; prejudice; stigma; mental health; wellbeing

For peer review only - http://bmjopen.bmj.com/site/about/guidelines.xhtml

Article Summary

- The use of the prospective United Kingdom Household Longitudinal Study allowed use to examine wellbeing over a 4 year period.
- The disability discrimination measure took into account several kinds of discriminatory behaviour and included multiple settings where perceived disability discrimination could be encountered.
- Our findings are based on perceptions of disability discrimination rather than objective encounters with disability discrimination.
- Disability discrimination was only assessed at one point in time, meaning our measure does not necessarily reflect pervasive discrimination.

Funding: This work was supported by the Economic and Social Research Council (https://esrc.ukri.org/), grant number ES/R005990/1. The funders had no role in study design, data collection and analysis, decision to publish, or preparation of the manuscript.

Competing interests: The authors declare that they have no conflict of interest.

Data sharing: The UKHLS datasets analysed during the current study are freely available in the UK Data Service repository https://ukdataservice.ac.uk/

For peer review only - http://bmjopen.bmj.com/site/about/guidelines.xhtml

Introduction

"Disability" is an umbrella term for long-term impairments, activity limitations and participation restrictions, experienced by an individual with a health condition in interaction with their environment[1–3]. In the UK, the prevalence of self-reported disability is rising, with 21% of the population reporting a disability in 2017-2018, an increase from 18% in 2007-2008[4]. This increase is likely driven by population ageing. The majority of disabled people report experiencing difficulties in everyday life. For example, disabled people in Britain have lower educational attainment, are less likely to be employed and are more likely to live in poverty than non-disabled adults[5], with knock on effects regarding access to health and other services[6].

These practical difficulties experienced by a person with a long-term impairment may be aggravated by and interact with discrimination in the person's environment. Disability discrimination has been defined as unwanted, exploitative or abusive conduct against disabled people which violates their dignity and security or creates an intimidating or offensive environment[7]. Although disability is a protected characteristic under equality legislation[1], disability discrimination is perceived to be common. In a 2015 population survey of 27,718 adults from 26 European countries, 50% of participants reported disability discrimination to be widespread, a 4% increase from 2012 data. However, the reasons behind this increase are unclear due to the cross-sectional nature of the Eurobarometer data[8].

Disability discrimination can happen in a variety of settings including on the street, in the workplace and in public venues such as shops or pubs[9]. Recent British data suggests that disabled people are more likely than non-disabled people to report feeling unsafe when walking alone and to worry about physical attack and theft [10,11]. Disability is the second most common motivator for hate crime incidents, after race in England and Wales[12]. In the

For peer review only - http://bmjopen.bmj.com/site/about/guidelines.xhtml

workplace, several studies suggest that a greater proportion of disabled than non-disabled individuals report experiences of discrimination [7,13].

A growing body of research has investigated discrimination as a determinant of wellbeing[14–16]. Meta-analyses have linked perceived discrimination with depression and psychological distress and with poorer life satisfaction and self-rated health[14–16]. However, disability discrimination was not assessed in two of these meta-analyses and the majority of previous working has focused on racism [14,15]. In the most recent pooled analysis of 328 studies, of which only 8 studies concerned physical illness discrimination and disability discrimination[16], the combined category of physical illness/disability discrimination was associated with greater psychological distress and lower self-esteem.

Several cross-sectional studies have assessed perceived disability discrimination alone in relation to wellbeing outcomes. One study of 229 individuals with an intellectual disability in England found that self-reported stigma was associated with a greater number of depression and anxiety symptoms[17]. Two analyses of the Australian Survey of Disability, Ageing and Carers (n=9655 and n=6183, respectively) linked disability discrimination with greater psychological distress[18,19]. This association was similarly observed in a Swedish general population survey[20]. Research has also linked perceived disability discrimination with lower life satisfaction in Canadian adolescents[21], Korean women with severe disabilities[22] and Israeli nationals with physical disabilities[23]. Perceived disability discrimination has been associated with poorer self-rated health cross-sectionally in four studies[19,24–26] including a general population analysis of 52,458 individuals, from the European Social Survey[25].

Cross-sectional correlations are difficult to interpret: perception of discrimination may result in emotional distress, but it is also possible that emotional distress leads to alterations in how people interpret social interactions with others. To date, only one study has assessed prospective associations between perceived disability discrimination and wellbeing [27]. In an

For peer review only - http://bmjopen.bmj.com/site/about/guidelines.xhtml

analysis of older adults (≥50 years) participating in the US-based Health and Retirement Study (HRS), perceived disability discrimination was associated with poorer life satisfaction, self-rated health, and greater loneliness over four-year follow-up.

Overall, previous research has been dominated by cross-sectional studies, precluding the assessment of the temporal relationship between perceived disability discrimination and wellbeing outcomes. No longitudinal studies have compared people with a disability who do or do not report discrimination and wellbeing outcomes. To address these limitations, this study aimed to investigate cross-sectional and prospective associations between perceived disability discrimination and wellbeing in a UK population cohort.

Methods

Study population

This study uses data from Understanding Society: The UK Household Longitudinal Study (UKHLS)[28]. The overarching purpose of UKHLS is to provide high quality longitudinal data about the health, work, education, income, family, and social life of the UK population [29]. Data collection began in 2009/10 (wave 1) with follow-ups annually. The current study uses data from waves 1 (2009/10) and 5 (2013/14). These data were collected through face-to-face interview via computer aided personal interview and self-completion paper questionnaires and from wave 3 via computer administered self-interview. The UKHLS comprises of a representative general population probability sample of UK households, in addition to an ethnic minority boost sample[29,30]. The general population sample is based on proportionality stratified clustered samples of residential addresses in England, Scotland and Wales. In Northern Ireland, an unclustered systematic random sample of domestic addresses was selected. The ethnic minority boost was selected from high concentration ethnic minority areas, where 80% of the United Kingdom's five major ethnic minorities live [29].

For peer review only - http://bmjopen.bmj.com/site/about/guidelines.xhtml

Our data come from the ‘extra 5 minutes sample’ of over 8,000 participants who had an additional 5 minutes of questions on issues pertinent to ethnicity research including discrimination. This sample comprises of mostly ethnic minorities (n = 6722) who were drawn from the ethnic minority boost along with a comparison group of white participants (n = 1428)[30]. We restricted our sample to those who responded to the disability discrimination questions (n = 4788) with a self-reported disability (n = 871). Self-reported disability was based on a positive response the question “*Do you have any health problems or disabilities which mean you have substantial difficulties with any of the following areas of your life”* across any of the 12 types of difficulty assessed. These included issues with manual dexterity and mobility, problems with memory or the ability to concentrate, difficulties with learning and understanding, as well as hearing and sight impairments. The response rates for the UKHLS general population sample and the ethnic minority boost at Wave 1 were 81.8% and72.4%, respectively [30]. The response rate for the “extra 5-min sample” was 42.5%. At wave 5 there was loss to follow-up (n=431), leaving a follow-up sample size of 440 participants. Our definition of disability did not include mental health-related impairments. Ethical approval for UKHLS was obtained from the University of Essex Ethics Committee. All participants provided fully informed consent.

Perceived disability discrimination

To assess discrimination, participants were asked whether in the past 12 months they (a) felt unsafe, (b) avoided going to or being in, (c) had been insulted, called names, threatened or shouted at, or (d) had been physically attacked, in seven different settings: 1) At school/college/work, 2) On public transport, 3) At or around bus or train stations, 4) In a taxi, 5) Public buildings such as shopping centres or pubs, 6) Outside on the street, in parks or other public places, or 7) At home. If they answered yes to any one of these questions, a follow-up

For peer review only - http://bmjopen.bmj.com/site/about/guidelines.xhtml

question asked them to choose a reason from a list of categories including disability, sex and ethnicity among others. It was possible to choose multiple settings and attributions for the perceived discrimination. Those who attributed any experience of discrimination to disability are treated as cases of perceived disability discrimination.

Outcome variables

Self-reported doctor-diagnosed clinical depression was measured at wave 1 (2009/10) with responses coded as yes/no. Depression was not analysed longitudinally due to a lack of incident cases. All other outcomes were assessed at waves 1 (2009/10) and 5 (2013/14). Psychological distress was measured using the General Health Questionnaire (GHQ)-12[31], which involved ratings of 12 statements including whether the individual had "*Been able to enjoy your normal day to day activities*" or whether they "*Felt constantly under strain*" with response options of 0="no" and 1="yes". Total scores range from 0 (least distressed) to 12 (most distressed). The 12-item short-form health survey (SF-12) mental component summary score was used to measure limitations caused by emotional, mental health and social functioning issues[32]. Items included ratings of feelings experienced over the past 4 weeks such as "*Have you felt downhearted or blue*?" or *"Accomplished less than you would like"*. Overall scores were derived using standard methods ranging from 0 (low functioning) to 100 (high functioning) [33]. Life satisfaction was assessed using one item asking participants how satisfied they were with their "life overall", with scores ranging from 1 (completely dissatisfied) to 7 (completely satisfied). Self-rated health was assessed using a single item: "*Would you say your health is…poor/fair/good/very good/excellent?"* In keeping with previous investigations [34,35] self-rated health was dichotomised with 0 being "good/very good/excellent" and 1 meaning "poor/fair".

For peer review only - http://bmjopen.bmj.com/site/about/guidelines.xhtml

Covariates

A number of covariates (assessed at wave 1) that are likely relevant to perceived disability discrimination and wellbeing were selected a priori for inclusion in our analyses. Age in years was entered as a continuous variable, as there may be age differences in reports of discrimination [36] and in wellbeing outcomes [37]. Sex was included as a binary variable (male/female) based on previous literature demonstrating sex differences in the impact of discrimination on health [38]. Income and education were included as covariates as there may be socio-economic differences in the perception of discrimination and in wellbeing outcomes [36,39]. Equivalised monthly household income was calculated by dividing total household net income by the modified Organization for Economic Cooperation and Development (OECD) equivalence scale to adjust for the effects of household size and composition[40]. Income was entered as a continuous variable in our models. Education was included as a 3-level categorical variable: 1 "university degree", 2 "high school qualification" and 3 "no qualification". As our sample was ethnically diverse, we included ethnicity as a 4-level variable with 1 being "white" including those of white British, white Irish and any other white background, 2 being "south Asian" including Indian, Pakistani and Bangladeshi individuals, 3 being "black" including black African and black Caribbean participants and 4 being "other" including individuals from Chinese and mixed backgrounds. There were 4 categories of impairment measured in the study: "physical" disability which included difficulties with manual dexterity and mobility; "cognitive" disability including problems with memory or the ability to concentrate, learn and understand; "sensory" disability including hearing (apart from using a standard hearing aid) and sight impairments (apart from wearing standard glasses) and "other" which encompassed reports of unspecified disability not captured in the other categories.

Statistical analyses

For peer review only - http://bmjopen.bmj.com/site/about/guidelines.xhtml

We compared the characteristics of those who did and those who did not report disability discrimination at wave 1 using Chi-squared tests for categorical variables and independent samples t-tests for continuous variables. Associations between perceived disability discrimination and the various wellbeing measures were assessed using linear regression for continuous outcomes and logistic regression for categorical outcomes. For cross-sectional analyses, depression, psychological distress (GHQ-12), SF-12 mental component score, life satisfaction and self-rated health at wave 1 (2009/10) were the outcome variables. For prospective analyses, psychological distress (GHQ-12), SF-12 mental component score, life satisfaction and self-rated health at wave 5 (2013/14) were the outcomes. Age, sex, household income, education, ethnicity and disability type at wave 1 were controlled for in all analyses. Baseline (wave 1) scores/status on the relevant wellbeing variable was included as an additional covariate in prospective analyses. Only those with complete case information at wave 1 (*n*=871) and wave 5 (*n*=440) were included in the analyses. Results from linear regression analyses are presented as unstandardized B and 95% confidence intervals (95% CI). Results from logistic regression analyses are presented as odds ratios (ORs) and 95% CI. All analyses were unweighted and conducted using SPSS v.24.

Sensitivity analyses

We carried out three sensitivity analyses. In our first, we assessed whether those who were lost to follow-up (*n* = 431) differed from those who provided data at both waves (*n* = 440). We tested whether this impacted the results by conducting the cross-sectional analyses (wave 1) including only those who provided follow-up data at wave 5. We carried out our second sensitivity analysis to test the possibility that one of the four types discriminatory behaviour contributing to the measure of perceived disability discrimination (i.e. feeling unsafe, avoiding somewhere, being insulted or being physically attacked) was driving the results. We tested this

For peer review only

cross-sectionally and prospectively by repeating our analyses removing each type of discriminatory behaviour in turn. In our third sensitivity analysis, we tested whether the prospective results from our complete cases analysis at wave 5 (n=440) were similar when missing outcome information was imputed for those participants lost to follow-up (n=431).

Results

A total of 871 participants were included in the study and of these 117 (13.4%) reported perceived disability discrimination. Disability discrimination was the mostly commonly reported form of discrimination in the sample, followed by age discrimination (4.3%), sex discrimination (3.9%), ethnicity discrimination (3.8%), religious discrimination (2.2%) and discrimination on the basis of sexual orientation (0.5%). Of the categories of disability discrimination assessed, the most commonly reported was feeing unsafe (86.1%; 95% CI 79.48-92.74), followed by avoiding somewhere (72.8%; 95% CI 64.08-81.55), being insulted (23.5%; 95% CI 14.33-32.73) and being physically attacked (2.8%; 95% CI 0.04-5.98). The most common settings in which disability discrimination was reported were on the street (77.8%; 95% CI 70.13-85.42), in public buildings such as shops or pubs (59.8%; 95% CI 50.81-68.84), on public transport (51.3%; 95% CI 42.09-60.47) and at or around bus or train stations (40.2%; 95% CI 31.16-49.19). A quarter of participants reported experiencing disability discrimination at home (25%; 95% CI 17.61-33.67). Perceived disability discrimination was less frequently reported in school or workplace settings (12.8%; 95% CI 6.67-18.97) or in taxis (12%; 95% CI 6-17.93). The prevalence of the various types of perceived disability discrimination and the settings in which the discrimination occurred for different types of disability can be found in Supplementary Table 1. There were no statistically significant

For peer review only - http://bmjopen.bmj.com/site/about/guidelines.xhtml

differences between people with different types of disability in discrimination type or discrimination setting.

The baseline characteristics of the sample are displayed in Table 1. The group who reported disability discrimination were younger on average (48.29 ± 14.89 years) than those who did not report discrimination (53.42 ± 16.56 years). They were more likely to be white (27.4% vs. 20.3%) and to be better educated than those who did not report discrimination, with a greater proportion holding university degrees (28.2% vs 22.7%). Physical disability was most common in those who did not perceive discrimination (46.6%), whereas other unspecified disabilities (65%) were most frequently reported by those who perceived discrimination.

Cross-sectional associations between perceived disability discrimination and wellbeing

Our findings suggest that individuals who perceived disability discrimination were significantly more likely to report a diagnosis of clinical depression (OR=5.40; 95% CI 3.25; 8.97, p <0.001) and were more likely to rate their health as fair/poor (OR=2.05; 95% CI 1.19; 3.51, p =0.009) than those who did not perceive disability discrimination, independent of covariates (first panel Table 2). Those who reported discrimination also had significantly higher levels of psychological distress (B = 3.28, 95% CI 2.41; 4.14, p <0.001), poorer mental functioning on the SF-12 (B = -7.35; 95% CI -9.70; -5.02, p <0.001) and lower life satisfaction (B = -1.27, 95% CI -1.66; -0.87, p <0.001), than those who did not report discrimination

Prospective associations between perceived disability discrimination and wellbeing

In prospective analyses (second panel Table 2), those who reported perceived disability discrimination at wave 1 had higher levels of psychological distress 4 years later at wave 5 than those who did not report discrimination, independent of covariates and baseline psychological distress (B = 2.88, 95% CI 1.39; 4.36, p <0.001). We detected a prospective association

For peer review only - http://bmjopen.bmj.com/site/about/guidelines.xhtml

between perceived disability discrimination at wave 1 and poorer SF-12 mental functioning at wave 5 (B = -5.12; 95% CI -8.91; -1.34, p =0.008). Those who reported disability discrimination at wave 1 had slightly lower life satisfaction (means= 4.14 vs 4.67) and a greater proportion rated their health as fair/poor (67.3% vs 62.1%) than those who did not report discrimination at follow-up (wave 5). However, these differences did not reach statistical significance.

Sensitivity analyses

In the first sensitivity analysis (Table 3), cross-sectional findings for those who provided complete data at wave 5 were similar to the full-sample at wave 1. The demographic characteristics of those lost to follow-up were similar to those of complete cases (Table 4). Only education differed significantly between the groups, with those who provided complete data at wave 5 more likely to hold a degree (27.0%) than those lost to follow-up (19.7%).

In the second sensitivity analysis, removing each of the discriminatory behaviours from the measure of discrimination in turn did not alter the cross-sectional results (Table 5). Prospectively, the association between perceived disability discrimination and increased psychological distress remained the same regardless of the type of discriminatory behaviour removed from the measure. For SF-12 mental functioning, the association was fairly robust to the type of discriminatory behaviour, but was slightly attenuated when "feeling unsafe" was removed from the discrimination variable (p = 0.058). Again, no significant prospective associations were detected for life satisfaction and self-rated health.

In our final sensitivity analysis (Supplementary Table 2), we repeated the prospective analyses with imputation for missing outcome information of participants lost to follow-up (n=431). The prospective relationship between perceived disability discrimination and poor SF-12 mental functioning remained (p = 0.034). However, there was no longer a statistically

For peer review only - http://bmjopen.bmj.com/site/about/guidelines.xhtml

significant prospective association between reported discrimination and psychological distress ($p = 0.128$).

Discussion

In a sample of UK-based participants with self-reported disability, perceived discrimination was associated with higher prevalence of depression, greater psychological distress, and poorer mental functioning, life satisfaction and self-rated health. Prospectively, disability discrimination was associated with increased psychological distress and worse mental functioning four-years later. Our results were robust to adjustment for a range of covariates and were not driven by any specific kind of discriminatory behaviour. No significant prospective relationships with life satisfaction and self-rated health were observed.

Previous literature has been dominated by cross-sectional studies. To our knowledge, only one previous study has investigated the prospective association between disability discrimination and wellbeing outcomes. In this analysis of US adults from the HRS cohort, perceived disability discrimination was associated with poorer life satisfaction and self-rated health over four year follow-up [27]. In the current study we observed poorer mental functioning and greater psychological distress four years later in those who reported disability discrimination, taking into account baseline scores on these variables. We failed to detect a significant association between perceived discrimination and life satisfaction or self-rated health at follow-up. Although on average, those who perceived disability discrimination in our sample had poorer life satisfaction and were more likely to rate their health as fair/poor at follow-up than those who did not perceive discrimination, these differences did not reach statistical significance. One reason for the divergence in findings between our study and the HRS analysis [27] may be study design. We limited our analyses to those with self-reported disability, whereas in the HRS study associations between wellbeing and disability

For peer review only - http://bmjopen.bmj.com/site/about/guidelines.xhtml

discrimination were assessed across the entire sample. Our analysis offers more precision in the assessment of the relationship between disability discrimination and wellbeing outcomes, by directly comparing people with disability who did and did not perceive discrimination. Another possibility for these null findings may be that significant associations between discrimination and life satisfaction and self-rated health do not become apparent until older adulthood, perhaps allowing for repeated exposures to disability discrimination. However, this assertion remains to be tested. Another potential explanation is that the impact of ongoing disability discrimination on life satisfaction and self-rated health in our sample had already become apparent at the time of the baseline survey, limiting the scope for further decline.

Our study adds to the cross-sectional literature linking perceived disability discrimination and poorer wellbeing outcomes by demonstrating associations in a community sample of disabled people living in the UK. Our results extend the findings of an earlier study linking stigma and depression in those with intellectual disability[17], by establishing this relationship in a sample with a broader range of disability. In keeping with previous studies, we observed greater psychological distress[18–20] and poorer life satisfaction[21–23,27] in those who reported disability discrimination. Our study adds to this existing evidence by demonstrating this link in a UK-based sample for the first time. Similar to earlier work from Australian, European and North American samples[19,24,27,41], we observed a relationship between perceived disability discrimination and poorer self-rated health. Cross-sectional studies cannot determine whether perceived disability discrimination predicts poor mental wellbeing, or whether perceptions of discrimination are an indicator of psychological distress. Our prospective findings therefore add to the field in establishing that perceived disability discrimination predicts psychological distress and poorer mental functioning, net of baseline associations, so has negative implications for future wellbeing.

For peer review only - http://bmjopen.bmj.com/site/about/guidelines.xhtml

This is an observational study and longitudinal analyses do not necessarily imply causality. There could be unmeasured factors responsible for the associations that emerged. Nevertheless, with regard to the pathways linking perceived disability discrimination and wellbeing, there are several possibilities that could explain our results. One mechanism could be that perceptions of disability discrimination in healthcare settings serve to impede access to health services. An analysis of HRS found that reports of frequent discrimination in healthcare settings were predictive of new or worsened disability over four year follow-up[42]. Quantitative[38,43] and qualitative[44] evidence suggests that those who perceive disability discrimination are less likely to seek healthcare. However, there may be sex differences in this association, with a Swedish study only detecting a relationship between disability discrimination and healthcare avoidance in women[38].However, no interaction between sex and perceived discrimination was detected in the current study (data not shown).

Poor health behaviours are another potential mechanism linking disability discrimination and poorer wellbeing. For example, perceived disability discrimination has been linked with worse sleep quality in the HRS, with psychological distress acting as a full mediator of this association[45]. It is possible that disabled people could engage in negative health behaviours as a means of coping with the psychological impact of discrimination. In a study of 304 individuals with disability, perceived disability discrimination was positively associated with illicit drug use[46]. Eating may offer a source of comfort in the face of discrimination[47]. A US study of over 5000 individuals, observed a link between physical disability discrimination and overeating[48].

Another possibility is that perceived disability discrimination and wellbeing are linked through disturbed stress-related biological processes. In line with the theory of allostatic load, perceived chronic discrimination causing frequent activation of the stress response system, could over time result in disturbances across multiple biological systems[49]. Systematic

For peer review only

reviews and meta-analyses, which have predominately focused on racism, suggest that discrimination is linked with heightened cardiovascular stress reactivity[14,50], while race[50–52] and weight discrimination[53] have been linked with alterations in cortisol. To our knowledge, no study has investigated associations between perceived disability discrimination and changes in cardiovascular or neuroendocrine activity. In the HRS cohort, perceived disability discrimination was linked with raised c-reactive protein levels cross-sectionally[54]. Heightened inflammation is thought to be predictive of poorer mental wellbeing[55], offering a plausible pathway between perceived disability discrimination and later psychological distress and poorer mental functioning seen in the present study. Further work is required to confirm this assertion, particularly as the HRS analysis was not limited to those with a confirmed disability.

Our study had several strengths. The use of the UKHLS cohort allowed us to examine wellbeing over a 4-year period across a wide age range (17 – 96 years), while adjusting statistically for factors that could confound associations. The discrimination measure took into account several kinds of discriminatory behaviour and included multiple settings where perceived disability discrimination could be encountered.

However, the study was not without limitations. We lost a considerable number of participants at follow-up, and although the cross-sectional findings did not differ between those who provided data at both waves and those lost to follow-up (sensitivity analysis 1), we cannot be sure that selection bias due to low retention did not impact our findings longitudinally due to the extent of missing data. Indeed, in imputed analyses (sensitivity analysis 3) the association between disability discrimination and wellbeing held for SF-12 mental functioning but not for psychological distress. Our findings are based on perceptions of disability discrimination rather than objective encounters with disability discrimination. It is possible that perceiving oneself as a target for discrimination and objective encounters with discrimination could have differing

For peer review only - http://bmjopen.bmj.com/site/about/guidelines.xhtml

consequences for wellbeing. Indeed, earlier work in a sample with significant health limitations, indicates that individuals with poorer mental wellbeing may be more likely to perceive stigma[56]. Future studies assessing reciprocal prospective associations between perceived disability discrimination and wellbeing could help to clarify this issue. Our discrimination measure was based on self-reports of experiences during the past 12 months and was therefore subject to recall bias. Further, this measure was not specific to disability discrimination. The fact that participants were able to attribute multiple reasons for their experience of discrimination, could have helped avoid priming or bias. Other tools specifically designed to assess disability discrimination could have garnered different results. Our sample was ethnically diverse, and we took ethnicity into account in our models. Although disability discrimination was the most commonly reported form of discrimination in this sample, perceived discrimination on the basis of ethnicity may also have been relevant for this sample. Further work is required to understand how disability discrimination interacts with ethnicity discrimination, as well as other types of discrimination to influence wellbeing. Disability discrimination was only assessed at one point in time, meaning our measure does not necessarily reflect pervasive discrimination. However, other work in UKHLS suggests that perceived disability discrimination is still frequently reported at later stages of data collection [57]. Future research is required to determine whether perceptions of disability discrimination are persistent or alter over time. We operationalised perceived discrimination as a simple binary variable and had no information on the frequency of encounters with discrimination over time. Therefore, the potential dose response relationship between the frequency of discrimination and wellbeing remains to be elucidated. Our study included participants with physical, cognitive and sensory disabilities. However, our sample is unlikely to have captured those with severe cognitive impairments due to the demands of survey participation. For a large proportion (37%) their disability type was unknown and classified as "other", limiting our

For peer review only

understanding. While, no one with a mental health-related disability was included in the physical, cognitive and sensory disabilities categories, we cannot be certain that the "other" category did not include participants with mental health-related impairments.

Overall, our study adds to the literature by demonstrating prospective associations between perceived disability discrimination and wellbeing outcomes. These findings emphasise the need to reduce the prevalence of disability discrimination, with the benefit of promoting equality as well as possible advantages for wellbeing too. Though complete elimination of disability discrimination is likely to be difficult, recognition of disability discrimination as an issue is the first step in preventing its occurrence. Addressing this could involve raising awareness through the use of campaigns. The Public Sector Equality Duty in the UK requires public bodies to have due regard to the need to eliminate discrimination and this awareness raising should begin early in life[7]. However, it is estimated that less than 40% of English primary schools have a disability equality scheme in place, with race and gender equality more often prioritised over disability equality[58]. Therefore, further effort on this issue is required[7], particularly as disability discrimination is perceived to be more widespread than gender discrimination in Europe[8].

As well as macro-level awareness raising, on an individual basis the negative impact of perceived disability discrimination on wellbeing may be buffered through the use of social support. In two cross-sectional studies of US adults with varied disability diagnoses, those with more friends reported greater life satisfaction and these friendships attenuated the link between functional impairment and poorer quality of life[59]. In an Israeli study, perceived disability discrimination and poorer life satisfaction were only linked in those with low and moderate levels of social support, with no association in those with greater levels of support[60]. Further research disability discrimination is necessary to develop awareness campaigns and to appropriately target individual-level interventions.

For peer review only - http://bmjopen.bmj.com/site/about/guidelines.xhtml

For peer review only

Author contributions: RAH conducted the statistical analysis and wrote the manuscript. AS edited and reviewed the manuscript. RPL edited and reviewed the manuscript. SJ provided scientific overview, edited and reviewed the manuscript.

Patient and Public Involvement

No patients or members of the public were involved in study design or conduct.

For peer review only

For peer review only - http://bmjopen.bmj.com/site/about/guidelines.xhtml

References

1 The Equality Act. 2010. https://www.legislation.gov.uk/ukpga/2010/15/contents

2 World Health Organisation. *World report on disability 2011*. World Health Organisation 2011.

3 Leonardi M, Bickenbach J, Ustun TB, *et al.* The definition of disability: what is in a name? *The Lancet* 2006;**368**:1219–21. doi:10.1016/S0140-6736(06)69498-1

4 Department for Work and Pensions. Family Resources Survey 2017-18. 2019.https://assets.publishing.service.gov.uk/government/uploads/system/uploads/attachment_data/file/790000/family-resources-survey-2017-18.pdf

5 Equality and Human Rights Commission. Being disabled in Britain: A journey less equal. 2017.

6 Office for National Statistics. Life Opportunities Survey: Wave 3 results. 2015.https://www.gov.uk/government/statistics/life-opportunities-survey-wave-3-results

7 Equality and Human Rights Commission. Hidden in plain sight: inquiry into disability-related harassment. 2011.https://www.equalityhumanrights.com/en/publication-download/hidden-plain-sight-inquiry-disability-related-harassment (accessed 28 Mar 2019).

8 European Union. *Discrimination in the EU in 2015*. 2015.

9 Sykes W, Groom C, Desai P. Disability-related harassment: the role of public bodies A qualitative research report. Manchester: : Equality and Human Rights Commission 2011.

10 Office for National Statistics. Crime Survey for England and Wales 2014-2015. 2016.

11 Scottish Government. Report of Independent Advisory Group on Hate Crime, Prejudice and Community Cohesion'. 2016.https://www.gov.scot/publications/report-independent-advisory-group-hate-crime-prejudice-community-cohesion/

12 Corcoran H, Lader D, Smith K. Hate crime, England and Wales, 2014 to 2015. 2015.

13 Coleman N, Sykes W, Groom C. Barriers to employment and unfair treatment at work: a quantitative analysis of disabled people's experiences. Equality and Human Rights Commission 2013.

14 Pascoe EA, Smart Richman L. Perceived discrimination and health: a meta-analytic review. *Psychol Bull* 2009;**135**:531–54. doi:10.1037/a0016059

15 Goto JB, Couto PFM, Bastos JL. [Systematic review of epidemiological studies on interpersonal discrimination and mental health]. *Cad Saude Publica* 2013;**29**:445–59.

16 Schmitt MT, Branscombe NR, Postmes T, *et al.* The consequences of perceived discrimination for psychological well-being: a meta-analytic review. -. *Psychol Bull* 2014;**140**:921–48.

For peer review only

17 Ali A, King M, Strydom A, *et al.* Self-reported stigma and symptoms of anxiety and depression in people with intellectual disabilities: Findings from a cross sectional study in England. *J Affect Disord* 2015;**187**:224–31. doi:10.1016/j.jad.2015.07.046

18 Temple JB, Kelaher M. Is disability exclusion associated with psychological distress? Australian evidence from a national cross-sectional survey. *BMJ Open* 2018;**8**:e020829. doi:10.1136/bmjopen-2017-020829

19 Krnjacki L, Priest N, Aitken Z, *et al.* Disability-based discrimination and health: findings from an Australian-based population study. *Aust N Z J Public Health* 2018;**42**:172–4.

20 Wamala S, Bostrom G, Nyqvist K. Perceived discrimination and psychological distress in Sweden. *Br J Psychiatry* 2007;**190**:75–6.

21 Daley A, Phipps S, Branscombe NR. The social complexities of disability: Discrimination, belonging and life satisfaction among Canadian youth. *SSM - Popul Health* 2018;**5**:55–63. doi:10.1016/j.ssmph.2018.05.003

22 Kim S. A Study of the Impact of Discrimination Experience on Life Satisfaction in Korean Women with Severe Disabilities. *World Acad Sci Eng Technol Int J Soc Behav Educ Econ Bus Ind Eng* 2015;**9**:3205–9.

23 Kagan M, Itzick M, Tal-Katz P. Demographic, Psychosocial, and Health- and Disability-Related Factors Associated With Psychological Distress Among People With Physical Disabilities. *Rehabil Psychol* 2018;**63**:392–9. doi:10.1037/rep0000206

24 Du Mont J, Forte T. Perceived discrimination and self-rated health in Canada: an exploratory study. *BMC Public Health* 2016;**16**:742. doi:10.1186/s12889-016-3344-y

25 Alvarez-Galvez J, Salvador-Carulla L. Perceived discrimination and self-rated health in Europe: evidence from the European Social Survey (2010). *PloS One* 2013;**8**:e74252. doi:10.1371/journal.pone.0074252

26 Emerson E. Self-reported exposure to disablism is associated with poorer self-reported health and well-being among adults with intellectual disabilities in England: A cross-sectional survey. *Public Health* 2010;**124**:682–9. doi:10.1016/j.puhe.2010.08.020

27 Sutin AR, Stephan Y, Carretta H, *et al.* Perceived discrimination and physical, cognitive, and emotional health in older adulthood. *Am J Geriatr Psychiatry Off J Am Assoc Geriatr Psychiatry* 2015;**23**:171–9. doi:10.1016/j.jagp.2014.03.007

28 University of Essex. Institute for Social and Economic Research, NatCen Social Research, Kantar Public. *Understanding Society: Waves 1-7, 2009-2016 and Harmonised BHPS: Waves 1-18, 1991-2009.* 9th Edition. UK data service 2017.

29 Knies G. *Understanding society: The UK household longitudinal study, Waves 1–7 (User Guide)*. University of Essex: : Colchester: Institute for Social and Economic Research 2017.

30 McFall S, Nandi A, Platt L. *Understanding Society: UK Household Longitudinal Study: User Guide to Ethnicity and Immigration Research*. 4th ed. Colchester: : University of Essex 2017.

For peer review only - http://bmjopen.bmj.com/site/about/guidelines.xhtml

31 Goldberg D, Williams P. *A user's guide to the General Health Questionnaire.* Basingstoke: : NFER-Nelson 1988.

32 Ware J, Kosinski M, Keller SD. A 12-Item Short-Form Health Survey: construction of scales and preliminary tests of reliability and validity. *Med Care* 1996;**34**:220–33.

33 Ware JE. *How to score version 2 of the SF-12 health survey (with a supplement documenting version 1) Edition: first*. Lincoln, RI: : Health Assessment Lab 2002.

34 Steptoe A, Jackson SE. The Life Skills of Older Americans: Association with Economic, Psychological, Social, and Health Outcomes. *Sci Rep* 2018;**8**:9669. doi:10.1038/s41598-018-27909-w

35 DeSalvo KB, Bloser N, Reynolds K, *et al.* Mortality prediction with a single general self-rated health question. A meta-analysis. *J Gen Intern Med* 2006;**21**:267–75. doi:10.1111/j.1525-1497.2005.00291.x

36 Hackett RA, Steptoe A, Jackson SE. Sex discrimination and mental health in women: A prospective analysis. *Health Psychol Off J Div Health Psychol Am Psychol Assoc* 2019;**38**:1014–24. doi:10.1037/hea0000796

37 World Health Organisation. *Depression and Other Common Mental Disorders: Global Health Estimates*. Geneva: : World Health Organisation 2017.

38 Wamala S, Merlo J, Boström G, *et al.* Perceived discrimination, socioeconomic disadvantage and refraining from seeking medical treatment in Sweden. *J Epidemiol Community Health* 2007;**61**:409–15. doi:10.1136/jech.2006.049999

39 Sareen J, Afifi TO, McMillan KA, *et al.* Relationship Between Household Income and Mental Disorders: Findings From a Population-Based Longitudinal Study. *Arch Gen Psychiatry* 2011;**68**:419–27. doi:10.1001/archgenpsychiatry.2011.15

40 OECD. *What are equivalence scales?* 2005.

41 Alvarez A, Faccioli J, Guinzbourg M, *et al.* Endocrine and inflammatory profiles in type 2 diabetic patients with and without major depressive disorder. *BMC Res Notes* 2013;**6**:61. doi:10.1186/1756-0500-6-61

42 Rogers SE, Thrasher AD, Miao Y, *et al.* Discrimination in Healthcare Settings is Associated with Disability in Older Adults: Health and Retirement Study, 2008-2012. *J Gen Intern Med* 2015;**30**:1413–20. doi:10.1007/s11606-015-3233-6

43 Moscoso-Porras MG, Alvarado GF. Association between perceived discrimination and healthcare-seeking behavior in people with a disability. *Disabil Health J* 2018;**11**:93–8. doi:10.1016/j.dhjo.2017.04.002

44 Ali A, Scior K, Ratti V, *et al.* Discrimination and other barriers to accessing health care: perspectives of patients with mild and moderate intellectual disability and their carers. *PloS One* 2013;**8**:e70855. doi:10.1371/journal.pone.0070855

For peer review only

For peer review only - http://bmjopen.bmj.com/site/about/guidelines.xhtml

45 Vaghela P, Sutin AR. Discrimination and sleep quality among older US adults: the mediating role of psychological distress. *Sleep Health J Natl Sleep Found* 2016;**2**:100–8. doi:10.1016/j.sleh.2016.02.003

46 Li Li DM. disability and illicit drug use: an application of labeling theory. *Deviant Behav* 2001;**22**:1–21. doi:10.1080/016396201750065784

47 Wansink B, Cheney MM, Chan N. Exploring comfort food preferences across age and gender. *Physiol Behav* 2003;**79**:739–47.

48 Sutin A, Robinson E, Daly M, *et al.* Weight discrimination and unhealthy eating-related behaviors. *Appetite* 2016;**102**:83–9. doi:10.1016/j.appet.2016.02.016

49 McEwen BS. Stress, adaptation, and disease. Allostasis and allostatic load. *Ann N Y Acad Sci* 1998;**840**:33–44.

50 Lockwood KG, Marsland AL, Matthews KA, *et al.* Perceived discrimination and cardiovascular health disparities: a multisystem review and health neuroscience perspective. *Ann N Y Acad Sci* 2018;**1428**:170–207. doi:10.1111/nyas.13939

51 Busse D, Yim IS, Campos B, *et al.* Discrimination and the HPA axis: current evidence and future directions. *J Behav Med* 2017;**40**:539–52. doi:10.1007/s10865-017-9830-6

52 Korous KM, Causadias JM, Casper DM. Racial discrimination and cortisol output: A meta-analysis. *Soc Sci Med 1982* 2017;**193**:90–100. doi:10.1016/j.socscimed.2017.09.042

53 Jackson SE, Kirschbaum C, Steptoe A. Perceived weight discrimination and chronic biochemical stress: A population-based study using cortisol in scalp hair. *Obes Silver Spring Md* 2016;**24**:2515–21. doi:10.1002/oby.21657

54 Sutin AR, Stephan Y, Luchetti M, *et al.* Perceived weight discrimination and C-reactive protein. *Obes Silver Spring Md* 2014;**22**:1959–61. doi:10.1002/oby.20789

55 Slavich GM, Irwin MR. From stress to inflammation and major depressive disorder: a social signal transduction theory of depression. *Psychol Bull* 2014;**140**:774–815. doi:10.1037/a0035302

56 Alonso J, Buron A, Bruffaerts R, *et al.* Association of perceived stigma and mood and anxiety disorders: results from the World Mental Health Surveys. *Acta Psychiatr Scand* 2008;**118**:305–14.

57 Emerson E, Krnjacki L, Llewellyn G, *et al.* Perceptions of safety and exposure to violence in public places among working age adults with disabilities or long-term health conditions in the UK: cross sectional study. *Public Health* 2016;**135**:91–6. doi:10.1016/j.puhe.2015.10.036

58 Beckett AE, Buckner L. Promoting positive attitudes towards disabled people: definition of, rationale and prospects for anti-disablist education. *Br J Sociol Educ* 2012;**33**:873–91. doi:10.1080/01425692.2012.692046

For peer review only - http://bmjopen.bmj.com/site/about/guidelines.xhtml

59 Silverman AM, Molton IR, Smith AE, *et al.* Solace in solidarity: Disability friendship networks buffer well-being. *Rehabil Psychol*;**62**:525–33.

60 Itzick M, Kagan M, Tal-Katz P. Perceived social support as a moderator between perceived discrimination and subjective well-being among people with physical disabilities in Israel. *Disabil Rehabil* 2018;**40**:2208–16. doi:10.1080/09638288.2017.1331380

For peer review only

1 The Equality Act. 2010. https://www.legislation.gov.uk/ukpga/2010/15/contents

2 World Health Organisation. *World report on disability 2011*. World Health Organisation 2011.

3 Leonardi M, Bickenbach J, Ustun TB, *et al.* The definition of disability: what is in a name? *The Lancet* 2006;**368**:1219–21. doi:10.1016/S0140-6736(06)69498-1

4 Department for Work and Pensions. Family Resources Survey 2017-18. 2019.https://assets.publishing.service.gov.uk/government/uploads/system/uploads/attachment_data/file/790000/family-resources-survey-2017-18.pdf

5 Equality and Human Rights Commission. Being disabled in Britain: A journey less equal. 2017.

6 Office for National Statistics. Life Opportunities Survey: Wave 3 results. 2015.https://www.gov.uk/government/statistics/life-opportunities-survey-wave-3-results

7 Equality and Human Rights Commission. Hidden in plain sight: inquiry into disability-related harassment. 2011.https://www.equalityhumanrights.com/en/publication-download/hidden-plain-sight-inquiry-disability-related-harassment (accessed 28 Mar 2019).

8 European Union. *Discrimination in the EU in 2015*. 2015.

9 Sykes W, Groom C, Desai P. Disability-related harassment: the role of public bodies A qualitative research report. Manchester: : Equality and Human Rights Commission 2011.

10 Office for National Statistics. Crime Survey for England and Wales 2014-2015. 2016.

11 Scottish Government. Report of Independent Advisory Group on Hate Crime, Prejudice and Community Cohesion'. 2016.https://www.gov.scot/publications/report-independent-advisory-group-hate-crime-prejudice-community-cohesion/

12 Corcoran H, Lader D, Smith K. Hate crime, England and Wales, 2014 to 2015. 2015.

13 Coleman N, Sykes W, Groom C. Barriers to employment and unfair treatment at work: a quantitative analysis of disabled people's experiences. Equality and Human Rights Commission 2013.

14 Pascoe EA, Smart Richman L. Perceived discrimination and health: a meta-analytic review. *Psychol Bull* 2009;**135**:531–54. doi:10.1037/a0016059

15 Goto JB, Couto PFM, Bastos JL. [Systematic review of epidemiological studies on interpersonal discrimination and mental health]. *Cad Saude Publica* 2013;**29**:445–59.

For peer review only - http://bmjopen.bmj.com/site/about/guidelines.xhtml

16 Schmitt MT, Branscombe NR, Postmes T, *et al.* The consequences of perceived discrimination for psychological well-being: a meta-analytic review. -. *Psychol Bull* 2014;**140**:921–48.

17 Ali A, King M, Strydom A, *et al.* Self-reported stigma and symptoms of anxiety and depression in people with intellectual disabilities: Findings from a cross sectional study in England. *J Affect Disord* 2015;**187**:224–31. doi:10.1016/j.jad.2015.07.046

18 Temple JB, Kelaher M. Is disability exclusion associated with psychological distress? Australian evidence from a national cross-sectional survey. *BMJ Open* 2018;**8**:e020829. doi:10.1136/bmjopen-2017-020829

19 Krnjacki L, Priest N, Aitken Z, *et al.* Disability-based discrimination and health: findings from an Australian-based population study. *Aust N Z J Public Health* 2018;**42**:172–4.

20 Wamala S, Bostrom G, Nyqvist K. Perceived discrimination and psychological distress in Sweden. *Br J Psychiatry* 2007;**190**:75–6.

21 Daley A, Phipps S, Branscombe NR. The social complexities of disability: Discrimination, belonging and life satisfaction among Canadian youth. *SSM - Popul Health* 2018;**5**:55–63. doi:10.1016/j.ssmph.2018.05.003

22 Kim S. A Study of the Impact of Discrimination Experience on Life Satisfaction in Korean Women with Severe Disabilities. *World Acad Sci Eng Technol Int J Soc Behav Educ Econ Bus Ind Eng* 2015;**9**:3205–9.

23 Kagan M, Itzick M, Tal-Katz P. Demographic, Psychosocial, and Health- and Disability-Related Factors Associated With Psychological Distress Among People With Physical Disabilities. *Rehabil Psychol* 2018;**63**:392–9. doi:10.1037/rep0000206

24 Du Mont J, Forte T. Perceived discrimination and self-rated health in Canada: an exploratory study. *BMC Public Health* 2016;**16**:742. doi:10.1186/s12889-016-3344-y

25 Alvarez-Galvez J, Salvador-Carulla L. Perceived discrimination and self-rated health in Europe: evidence from the European Social Survey (2010). *PloS One* 2013;**8**:e74252. doi:10.1371/journal.pone.0074252

26 Emerson E. Self-reported exposure to disablism is associated with poorer self-reported health and well-being among adults with intellectual disabilities in England: A cross-sectional survey. *Public Health* 2010;**124**:682–9. doi:10.1016/j.puhe.2010.08.020

27 Sutin AR, Stephan Y, Carretta H, *et al.* Perceived discrimination and physical, cognitive, and emotional health in older adulthood. *Am J Geriatr Psychiatry Off J Am Assoc Geriatr Psychiatry* 2015;**23**:171–9. doi:10.1016/j.jagp.2014.03.007

28 University of Essex. Institute for Social and Economic Research, NatCen Social Research, Kantar Public. *Understanding Society: Waves 1-7, 2009-2016 and Harmonised BHPS: Waves 1-18, 1991-2009.* 9th Edition. UK data service 2017.

29 Knies G. *Understanding society: The UK household longitudinal study, Waves 1–7 (User Guide)*. University of Essex: : Colchester: Institute for Social and Economic Research 2017.

For peer review only - http://bmjopen.bmj.com/site/about/guidelines.xhtml

30 McFall S, Nandi A, Platt L. *Understanding Society: UK Household Longitudinal Study: User Guide to Ethnicity and Immigration Research*. 4th ed. Colchester: : University of Essex 2017.

31 Goldberg D, Williams P. *A user's guide to the General Health Questionnaire.* Basingstoke: : NFER-Nelson 1988.

32 Ware J, Kosinski M, Keller SD. A 12-Item Short-Form Health Survey: construction of scales and preliminary tests of reliability and validity. *Med Care* 1996;**34**:220–33.

33 Ware JE. *How to score version 2 of the SF-12 health survey (with a supplement documenting version 1) Edition: first*. Lincoln, RI: : Health Assessment Lab 2002.

34 Steptoe A, Jackson SE. The Life Skills of Older Americans: Association with Economic, Psychological, Social, and Health Outcomes. *Sci Rep* 2018;**8**:9669. doi:10.1038/s41598-018-27909-w

35 DeSalvo KB, Bloser N, Reynolds K, *et al.* Mortality prediction with a single general self-rated health question. A meta-analysis. *J Gen Intern Med* 2006;**21**:267–75. doi:10.1111/j.1525-1497.2005.00291.x

36 Hackett RA, Steptoe A, Jackson SE. Sex discrimination and mental health in women: A prospective analysis. *Health Psychol Off J Div Health Psychol Am Psychol Assoc* 2019;**38**:1014–24. doi:10.1037/hea0000796

37 World Health Organisation. *Depression and Other Common Mental Disorders: Global Health Estimates*. Geneva: : World Health Organisation 2017.

38 Wamala S, Merlo J, Boström G, *et al.* Perceived discrimination, socioeconomic disadvantage and refraining from seeking medical treatment in Sweden. *J Epidemiol Community Health* 2007;**61**:409–15. doi:10.1136/jech.2006.049999

39 Sareen J, Afifi TO, McMillan KA, *et al.* Relationship Between Household Income and Mental Disorders: Findings From a Population-Based Longitudinal Study. *Arch Gen Psychiatry* 2011;**68**:419–27. doi:10.1001/archgenpsychiatry.2011.15

40 OECD. *What are equivalence scales?* 2005.

41 Alvarez A, Faccioli J, Guinzbourg M, *et al.* Endocrine and inflammatory profiles in type 2 diabetic patients with and without major depressive disorder. *BMC Res Notes* 2013;**6**:61. doi:10.1186/1756-0500-6-61

42 Rogers SE, Thrasher AD, Miao Y, *et al.* Discrimination in Healthcare Settings is Associated with Disability in Older Adults: Health and Retirement Study, 2008-2012. *J Gen Intern Med* 2015;**30**:1413–20. doi:10.1007/s11606-015-3233-6

43 Moscoso-Porras MG, Alvarado GF. Association between perceived discrimination and healthcare-seeking behavior in people with a disability. *Disabil Health J* 2018;**11**:93–8. doi:10.1016/j.dhjo.2017.04.002

For peer review only - http://bmjopen.bmj.com/site/about/guidelines.xhtml

44 Ali A, Scior K, Ratti V, *et al.* Discrimination and other barriers to accessing health care: perspectives of patients with mild and moderate intellectual disability and their carers. *PloS One* 2013;**8**:e70855. doi:10.1371/journal.pone.0070855

45 Vaghela P, Sutin AR. Discrimination and sleep quality among older US adults: the mediating role of psychological distress. *Sleep Health J Natl Sleep Found* 2016;**2**:100–8. doi:10.1016/j.sleh.2016.02.003

46 Li Li DM. disability and illicit drug use: an application of labeling theory. *Deviant Behav* 2001;**22**:1–21. doi:10.1080/016396201750065784

47 Wansink B, Cheney MM, Chan N. Exploring comfort food preferences across age and gender. *Physiol Behav* 2003;**79**:739–47.

48 Sutin A, Robinson E, Daly M, *et al.* Weight discrimination and unhealthy eating-related behaviors. *Appetite* 2016;**102**:83–9. doi:10.1016/j.appet.2016.02.016

49 McEwen BS. Stress, adaptation, and disease. Allostasis and allostatic load. *Ann N Y Acad Sci* 1998;**840**:33–44.

50 Lockwood KG, Marsland AL, Matthews KA, *et al.* Perceived discrimination and cardiovascular health disparities: a multisystem review and health neuroscience perspective. *Ann N Y Acad Sci* 2018;**1428**:170–207. doi:10.1111/nyas.13939

51 Busse D, Yim IS, Campos B, *et al.* Discrimination and the HPA axis: current evidence and future directions. *J Behav Med* 2017;**40**:539–52. doi:10.1007/s10865-017-9830-6

52 Korous KM, Causadias JM, Casper DM. Racial discrimination and cortisol output: A meta-analysis. *Soc Sci Med 1982* 2017;**193**:90–100. doi:10.1016/j.socscimed.2017.09.042

53 Jackson SE, Kirschbaum C, Steptoe A. Perceived weight discrimination and chronic biochemical stress: A population-based study using cortisol in scalp hair. *Obes Silver Spring Md* 2016;**24**:2515–21. doi:10.1002/oby.21657

54 Sutin AR, Stephan Y, Luchetti M, *et al.* Perceived weight discrimination and C-reactive protein. *Obes Silver Spring Md* 2014;**22**:1959–61. doi:10.1002/oby.20789

55 Slavich GM, Irwin MR. From stress to inflammation and major depressive disorder: a social signal transduction theory of depression. *Psychol Bull* 2014;**140**:774–815. doi:10.1037/a0035302

56 Alonso J, Buron A, Bruffaerts R, *et al.* Association of perceived stigma and mood and anxiety disorders: results from the World Mental Health Surveys. *Acta Psychiatr Scand* 2008;**118**:305–14.

57 Emerson E, Krnjacki L, Llewellyn G, *et al.* Perceptions of safety and exposure to violence in public places among working age adults with disabilities or long-term health conditions in the UK: cross sectional study. *Public Health* 2016;**135**:91–6. doi:10.1016/j.puhe.2015.10.036

For peer review only

58 Beckett AE, Buckner L. Promoting positive attitudes towards disabled people: definition of, rationale and prospects for anti-disablist education. *Br J Sociol Educ* 2012;**33**:873–91. doi:10.1080/01425692.2012.692046

59 Silverman AM, Molton IR, Smith AE, *et al.* Solace in solidarity: Disability friendship networks buffer well-being. *Rehabil Psychol*;**62**:525–33.

60 Itzick M, Kagan M, Tal-Katz P. Perceived social support as a moderator between perceived discrimination and subjective well-being among people with physical disabilities in Israel. *Disabil Rehabil* 2018;**40**:2208–16. doi:10.1080/09638288.2017.1331380

For peer review only

For peer review only - http://bmjopen.bmj.com/site/about/guidelines.xhtml

Table 1 Associations between perceived disability discrimination and sociodemographic factors (wave 1) and wellbeing measures (waves 1 and 5)

	Overall sample (*n*= 871)	No perceived discrimination (*n* = 754)	Perceived discrimination (*n* = 117)	*p*
Age (years)	52.73 (16.43)	53.42 (16.56)	48.29 (14.89)	0.001
17-34	120 (13.7%)	100 (13.3%)	20 (17.1%)	
35-44	172 (19.7%)	149 (19.8%)	23 (19.7%)	
45-54	184 (21.1%)	150 (19.9%)	34 (29.1%)	
55-64	157 (18.0%)	134 (17.8%)	23 (19.7%)	
65+	238 (27.3%)	221 (29.3%)	17 (14.5%)	
Sex (% men)	388 (44.5%)	334 (44.3%)	54 (46.2%)	0.707
Household income (£)	1118.42 (902.54)	1123.28 (930.47)	1087.07 (698.65)	0.687
£0-499	117 (13.4%)	105 (13.9%)	12 (10.3%)	
£500-999	369 (42.4%)	314 (41.6%)	55 (47.0%)	
£1000-1499	216 (24.8%)	189 (25.1%)	27 (23.1%)	
£1500-1999	90 (10.3%)	77 (10.2%)	13 (11.1%)	
£2000+	79 (9.1%)	69 (9.2%)	10 (8.5%)	
Education (% yes)				0.003
University Degree	204 (23.4%)	171 (22.7%)	33 (28.2%)	-
School qualification	342 (39.3%)	285 (37.8%)	57 (48.7%)	-
No qualification	325 (37.3%)	298 (39.5%)	27 (23.1%)	-
Ethnicity				0.002
White	185 (21.2%)	153 (20.3%)	32 (27.4%)	-
South Asian	369 (42.4%)	333 (44.2%)	36 (30.8%)	-
Black	189 (21.7%)	168 (22.3%)	21 (17.9%)	-
Other	128 (14.7%)	100 (13.3%)	28 (23.9%)	-
Disability type				< 0.001
Physical	367 (42.1%)	351 (46.6%)	16 (13.7%)	
Sensory	95 (10.9%)	84 (11.1%)	11 (9.4%)	
Cognitive	87 (10.0%)	73 (9.7%)	14 (12.0%)	
Other	322 (37.0%)	246 (32.6%)	76 (65.0%)	
Wellbeing measures (wave 1)				
Depression	115 (13.2%)	65 (8.7%)	50 (42.7%)	< 0.001
Psychological distress	3.43 (3.85)	2.85 (3.49)	6.65 (4.18)	< 0.001
SF-12 mental	43.87 (12.61)	45.16 (12.22)	35.72 (12.02)	< 0.001
Life satisfaction	4.49 (1.71)	4.71 (1.63)	3.29 (1.67)	< 0.001
Fair/poor self-rated health	620 (71.2%)	525 (69.6%)	95 (81.2%)	0.010
Wellbeing measures (wave 5)				
Psychological distress	3.33 (3.99)	2.86 (3.82)	5.98 (3.91)	< 0.001
SF-12 mental	44.38 (12.20)	45.79 (11.62)	36.47 (12.51)	< 0.001
Life satisfaction	4.48 (1.61)	4.62 (1.57)	3.67 (1.64)	< 0.001
Fair/poor self-rated health	276 (62.7%)	235 (61.0%)	41 (74.5%)	0.053

Data are presented as means (SD) and n (%). Percentages are valid percent.

For peer review only - http://bmjopen.bmj.com/site/about/guidelines.xhtml

Table 2: Cross-sectional and prospective associations between perceived disability discrimination and wellbeing outcomes

	**Depression** OR (95% CI)	**Psychological distress** *B* (95% CI)	**SF-12 mental** *B* (95% CI)	**Life satisfaction** *B* (95% CI)	**Fair/poor self-rated health** *B* (95% CI)
**Wave 1**					
No perceived discrimination	1 (Reference)[a]	Reference[b]	Reference[c]	Reference[d]	1 (Reference)[e]
Perceived discrimination	5.40 [3.25; 8.97]***	3.28 [2.41; 4.14]***	-7.35 [-9.70; -5.02]***	-1.27 [-1.66; -0.87]***	2.05 [1.19; 3.51]**
**Wave 5**					
No perceived discrimination	-	Reference[f]	Reference[g]	Reference[h]	1 (Reference)[i]
Perceived discrimination	-	2.88 [1.39; 4.36]***	-5.12 [-8.91; -1.34]**	-0.53 [-1.18; 0.11]	1.29 [0.59; 2.83]

All analyses are adjusted for age, sex, household income, education, ethnicity and disability type. Prospective analyses are additionally adjusted for baseline wellbeing status/score.

[a]= *n*= 751 for the no perceived discrimination group; *n*= 117 for the perceived discrimination group; [b]= *n*= 454 for the no perceived discrimination group; *n*= 82 for the perceived discrimination group; [c]= *n*= 742 for the no perceived discrimination group; *n*= 117 for the perceived discrimination group; [d]= *n*= 454 for the no perceived discrimination group; *n*= 84 for the perceived discrimination group; [e]= *n*= 754 for the no perceived discrimination group; *n*= 117 for the perceived discrimination group; [f]= *n*= 177 for the no perceived discrimination group; *n*= 31 for the perceived discrimination group; [g]= *n*= 239 for the no perceived discrimination group; *n*= 43 for the perceived discrimination group; [h]= *n*= 171 for the no perceived discrimination group; *n*= 34 for the perceived discrimination group; [i]= *n*= 385 for the no perceived discrimination group; *n*= 55 for the perceived discrimination group.

CI = confidence interval, OR = odds ratio

*p<0.05, **p<0.01, ***p <0.001

Possible scores on the psychological distress scale range from 0-12, SF-12 mental component scale range from 0-100 and the life satisfaction scale scores range from 0-7.

For peer review only - http://bmjopen.bmj.com/site/about/guidelines.xhtml

Table 3: Cross-sectional and prospective associations between perceived disability discrimination and wellbeing outcomes (complete cases at wave 5)

	**Psychological distress**[a] *B* (95% CI)	**SF-12 mental**[b] *B* (95% CI)	**Life satisfaction**[c] *B* (95% CI)	**Fair/poor self-rated health**[d] *B* (95% CI)
**Wave 1**				
No perceived discrimination	Reference	Reference	Reference	1 (Reference)
Perceived discrimination	2.65 [1.21; 4.08]***	-7.20 [-11.01; -3.39]***	-1.27 [-1.91; -0.63]***	2.66 [1.16; 6.08]*
**Wave 5**				
No perceived discrimination	Reference	Reference	Reference	1 (Reference)
Perceived discrimination	2.88 [1.39; 4.36]***	-5.12 [-8.91; -1.34]**	-0.53 [-1.18; 0.11]	1.29 [0.59; 2.83]

All analyses are adjusted for age, sex, household income, education, ethnicity and disability type. Prospective analyses are additionally adjusted for baseline wellbeing status/score.

[a]= *n*= 177 for the no perceived discrimination group; *n*= 31 for the perceived discrimination group; [b]= *n*= 239 for the no perceived discrimination group; *n*= 43 for the perceived discrimination group; [c]= *n*= 171 for the no perceived discrimination group; *n*= 34 for the perceived discrimination group; [d]= *n*= 385 for the no perceived discrimination group; *n*= 55 for the perceived discrimination group.

CI = confidence interval, OR = odds ratio

*p<0.05, **p<0.01, ***p <0.001

Possible scores on the psychological distress scale range from 0-12, SF-12 mental component scale range from 0-100 and the life satisfaction scale scores range from 0-7.

For peer review only

Table 4 Participant characteristics at wave 1 (2009/10) of complete cases and those lost to follow-up

	Lost to follow-up (n=431)	Complete cases (n=440)	*p*
Age (years)	52.26 (17.08)	53.19 (15.76)	0.407
17-34	25 (5.8%)	12 (2.7%)	
35-44	46 (10.7%)	37 (8.4%)	
45-54	79 (18.4%)	93 (21.1%)	
55-64	91 (21.2%)	93 (21.1%)	
65+	189 (44.0%)	205 (46.6%)	
Sex (% men)	200 (46.4%)	188 (42.7%)	0.275
Household income (£)	1101.26 (1037.11)	1135.22 (748.46)	0.579
£0-499	69 (16.0%)	48 (10.9%)	
£500-999	175 (40.6%)	194 (44.1%)	
£1000-1499	113 (26.2%)	103 (23.4%)	
£1500-1999	42 (9.7%)	48 (10.9%)	
£2000+	32 (7.4%)	47 (10.7%)	
Education (% yes)			0.024
University Degree	85 (19.7%)	119 (27.0%)	-
School qualification	171 (39.7%)	171 (38.9%)	-
No qualification	175 (40.6%)	150 (34.1%)	-
Ethnicity			0.213
White	88 (20.4%)	97 (22.0%)	-
South Asian	172 (39.9%)	197 (44.8%)	-
Black	105 (24.4%)	84 (19.1%)	-
Other	66 (15.3%)	62 (14.1%)	-
Disability type			0.189
Physical	166 (38.5%)	201 (45.7%)	
Sensory	51 (11.8%)	44 (10.0%)	
Cognitive	47 (10.9%)	40 (9.1%)	
Other	167 (38.7%)	155 (35.2%)	
Mental wellbeing			
Psychological distress	3.46 (3.85)	3.41 (3.86)	0.874
SF-12	43.72 (12.93)	44.01 (12.30)	0.741
Life satisfaction	4.40 (1.75)	4.56 (1.69)	0.306
Self-rated health (% fair/poor)	319 (74.0%)	301 (68.4%)	0.068

For peer review only

For peer review only - http://bmjopen.bmj.com/site/about/guidelines.xhtml

Data are presented as means (SD) and n (%)
*Complete cases are defined as those who were present at wave 1 and provided data on at least one wellbeing measure at wave 5.

For peer review only

For peer review only - http://bmjopen.bmj.com/site/about/guidelines.xhtml

Table 5: Sensitivity analysis: Perceived disability discrimination measure excluding each discriminatory behaviour in turn

Cross-sectional analyses (wave 1)		Model 1	Model 2	Model 3	Model 4
Depression	OR [95%CI]	4.41 (2.55;7.60)***	5.24 (3.06;8.98)***	4.80 (2.92;7.88)***	5.41 (3.26;8.98)***
Psychological distress	Coeff. [95%CI]	3.64 (2.68;4.60)***	3.28 (2.33;4.23)***	3.13 (2.26;4.01)***	3.27 (2.41;4.14)***
SF-12 mental	Coeff. [95%CI]	-6.63 (-9.29;-3.97)***	-7.61 (-10.15;-5.08)***	-7.53 (-9.89;-5.18)***	-7.35 (-9.68;-5.02)***
Life satisfaction	Coeff. [95%CI]	-1.23 (-1.69;-0.76)***	-1.45 (-1.88;-1.02)***	-1.24 (-1.64;-0.85)***	-1.27 (-1.66;-0.87)***
Fair/poor self-rated health	OR [95%CI]	1.92 (1.04;3.53)*	2.39 (1.32;4.33)**	2.21 (1.28;3.81)**	2.04 (1.19;3.50)**
**Prospective analyses (wave 5)**		**Model 1**	**Model 2**	**Model 3**	**Model 4**
Psychological distress	Coeff. [95%CI]	2.78 (1.14;4.41)***	2.69 (1.15;4.24)***	2.89 (1.45;4.33)***	2.88 (1.39;4.36)***
SF-12 mental	Coeff. [95%CI]	-4.33 (-8.81;0.14)	-4.90 (-8.86;-0.94)**	-5.94 (-9.59;-2.28)**	-5.13 (-8.91;-1.34)**
Life satisfaction	Coeff. [95%CI]	-0.39 (-1.14;0.36)	-0.37 (-1.06;0.32)	-0.39 (-1.02;0.25)	-0.53 (-1.18;0.11)
Fair/poor self-rated health	OR [95%CI]	1.23 (0.50;3.03)	1.30 (0.57;2.95)	1.31 (0.61;2.83)	1.29 (0.59;2.83)

All analyses are adjusted for age, sex, household income, education, ethnicity and disability type. Prospective analyses are additionally adjusted for baseline wellbeing status/score.
Model 1 excludes "*felt unsafe at some place*" from the measure of perceived gender discrimination; Model 2 excludes "*avoided some place* "; Model 3 excludes ""*was insulted at some place* "; and Model 4 excludes "*was attacked at some place*"
Coeff = unstandardized B coefficient (white rows), CI = confidence interval, OR = odds ratio (grey rows), SE = standard error.
**p*<0.05, ***p*<0.01, ****p*<0.001
Possible scores on the psychological distress scale range from 0-12, SF-12 mental component scale range from 0-100 and the life satisfaction scale scores range from 0-7.

For peer review only - http://bmjopen.bmj.com/site/about/guidelines.xhtml

Supplementary table 1: Perceived disability discrimination types and settings by type of disability

**Types of discrimination**	**Physical**	**Sensory**	**Cognitive**	**Other**	***p* value**
Felt unsafe at someplace	12 (85.7%)	9 (90%)	12 (92.3%)	60 (84.5%)	0.874
Avoided at someplace	8 (53.3%)	6 (75%)	9 (75%)	52 (69.3%)	0.336
Felt insulted at someplace	3 (25%)	1 (12.5%)	1 (10%)	15 (27.3%)	0.572
Attacked at someplace	0 (0%)	0 (0%)	0(0%)	3(4.4%)	0.621
**Settings**					
School/work	2 (12.5%)	1 (9.1%)	0 (0%)	12 (15.8%)	0.424
Public transport	10 (62.5%)	3 (27.3%)	6 (42.9%)	41 (53.9%)	0.266
Bus or train stations	5 (31.3%)	2 (18.2%)	6 (42.9%)	34 (44.7%)	0.328
Taxis	4 (25%)	0 (0%)	1 (7.1%)	9 (11.8%)	0.223
Public buildings	8 (50%)	7 (63.6%)	7 (50%)	48 (63.2%)	0.654
On the street	12 (75%)	8 (72.7%)	9 (64.3%)	62 (81.6%)	0.504
At home	3 (18.8%)	3 (27.3%)	4 (28.6%)	20 (26.3%)	0.920

Data are presented as numbers (% yes)

For peer review only

Supplementary table 2: Prospective associations between perceived disability discrimination and wellbeing outcomes (imputed analysis)

	Psychological distress[a]	SF-12 mental[b]	Life satisfaction[c]	Fair/poor self-rated health[d]
	B (95% CI)	*B* (95% CI)	*B* (95% CI)	*B* (95% CI)
**Wave 5**				
No perceived discrimination	Reference	Reference	Reference	1 (Reference)
Perceived discrimination	0.22 [-3.21; 3.64]	-4.55 [-8.75; -0.34]*	-0.49 [-1.10; 0.12]	1.38 [0.65; 2.93]

All analyses are adjusted for age, sex, household income, education, ethnicity and disability type. Prospective analyses are additionally adjusted for baseline wellbeing status/score.

[a]= *n*= 454 for the no perceived discrimination group; *n*= 82 for the perceived discrimination group; [b]= *n*= 742 for the no perceived discrimination group; *n*= 117 for the perceived discrimination group; [c]= *n*= 454 for the no perceived discrimination group; *n*= 84 for the perceived discrimination group; [d]= *n*= 754 for the no perceived discrimination group; *n*= 117 for the perceived discrimination group

*p<0.05, **p<0.01, ***p <0.001

CI = confidence interval, OR = odds ratio

Possible scores on the psychological distress scale range from 0-12, SF-12 mental component scale range from 0-100 and the life satisfaction scale scores range from 0-7.

For peer review only

For peer review only

Reporting checklist for cohort study.

Based on the STROBE cohort guidelines.

Instructions to authors

Complete this checklist by entering the page numbers from your manuscript where readers will find each of the items listed below.

Your article may not currently address all the items on the checklist. Please modify your text to include the missing information. If you are certain that an item does not apply, please write "n/a" and provide a short explanation.

Upload your completed checklist as an extra file when you submit to a journal.

In your methods section, say that you used the STROBE cohortreporting guidelines, and cite them as:

von Elm E, Altman DG, Egger M, Pocock SJ, Gotzsche PC, Vandenbroucke JP. The Strengthening the Reporting of Observational Studies in Epidemiology (STROBE) Statement: guidelines for reporting observational studies.

		Reporting Item	Page Number
**Title and abstract**			
Title	#1a	Indicate the study's design with a commonly used term in the title or the abstract	1
Abstract	#1b	Provide in the abstract an informative and balanced summary	2

For peer review only

		of what was done and what was found	
**Introduction**			
Background / rationale	#2	Explain the scientific background and rationale for the investigation being reported	4
Objectives	#3	State specific objectives, including any prespecified hypotheses	6
**Methods**			
Study design	#4	Present key elements of study design early in the paper	6
6Setting	#5	Describe the setting, locations, and relevant dates, including periods of recruitment, exposure, follow-up, and data collection	6
Eligibility criteria	#6a	Give the eligibility criteria, and the sources and methods of selection of participants. Describe methods of follow-up.	6
Eligibility criteria	#6b	For matched studies, give matching criteria and number of exposed and unexposed	N/A
Variables	#7	Clearly define all outcomes, exposures, predictors, potential confounders, and effect modifiers. Give diagnostic criteria, if applicable	7-8
Data sources / measurement	#8	For each variable of interest give sources of data and details of methods of assessment (measurement). Describe comparability of assessment methods if there is more than one group. Give information separately for for exposed and unexposed groups if applicable.	7-8

For peer review only

Bias	#9	Describe any efforts to address potential sources of bias	8
Study size	#10	Explain how the study size was arrived at	6
Quantitative variables	#11	Explain how quantitative variables were handled in the analyses. If applicable, describe which groupings were chosen, and why	9
Statistical methods	#12a	Describe all statistical methods, including those used to control for confounding	9
Statistical methods	#12b	Describe any methods used to examine subgroups and interactions	9
Statistical methods	#12c	Explain how missing data were addressed	6
Statistical methods	#12d	If applicable, explain how loss to follow-up was addressed	6
Statistical methods	#12e	Describe any sensitivity analyses	9-10
**Results**			
Participants	#13a	Report numbers of individuals at each stage of study—eg numbers potentially eligible, examined for eligibility, confirmed eligible, included in the study, completing follow-up, and analysed. Give information separately for for exposed and unexposed groups if applicable.	6
Participants	#13b	Give reasons for non-participation at each stage	N/A

For peer review only

Participants	#13c	Consider use of a flow diagram	N/A
Descriptive data	#14a	Give characteristics of study participants (eg demographic, clinical, social) and information on exposures and potential confounders. Give information separately for exposed and unexposed groups if applicable.	10
Descriptive data	#14b	Indicate number of participants with missing data for each variable of interest	6
Descriptive data	#14c	Summarise follow-up time (eg, average and total amount)	6
Outcome data	#15	Report numbers of outcome events or summary measures over time. Give information separately for exposed and unexposed groups if applicable.	N/A
Main results	#16a	Give unadjusted estimates and, if applicable, confounder-adjusted estimates and their precision (eg, 95% confidence interval). Make clear which confounders were adjusted for and why they were included	11
Main results	#16b	Report category boundaries when continuous variables were categorized	9
Main results	#16c	If relevant, consider translating estimates of relative risk into absolute risk for a meaningful time period	N/A
Other analyses	#17	Report other analyses done—e.g., analyses of subgroups and interactions, and sensitivity analyses	12

Discussion

For peer review only

Key results	#18	Summarise key results with reference to study objectives	12
Limitations	#19	Discuss limitations of the study, taking into account sources of potential bias or imprecision. Discuss both direction and magnitude of any potential bias.	16
Interpretation	#20	Give a cautious overall interpretation considering objectives, limitations, multiplicity of analyses, results from similar studies, and other relevant evidence.	12-14
Generalisability	#21	Discuss the generalisability (external validity) of the study results	16-17

Other Information

Funding	#22	Give the source of funding and the role of the funders for the present study and, if applicable, for the original study on which the present article is based	1

None The STROBE checklist is distributed under the terms of the Creative Commons Attribution License CC-BY. This checklist can be completed online using https://www.goodreports.org/, a tool made by the EQUATOR Network in collaboration with Penelope.ai

For peer review only
